# Population and Landscape Genetics Provide Insights Into Species Conservation of Two Evergreen Oaks in Qinghai–Tibet Plateau and Adjacent Regions

**DOI:** 10.3389/fpls.2022.858526

**Published:** 2022-05-19

**Authors:** Keke Liu, Min Qi, Fang K. Du

**Affiliations:** School of Ecology and Nature Conservation, Beijing Forestry University, Beijing, China

**Keywords:** species conservation, migration routes, genotype-environment association, *Quercus aquifolioides*, *Quercus spinosa*, Hengduan Mountains, Qinling Mountains

## Abstract

The combination of population and landscape genetics can facilitate the understanding of conservation strategy under the changing climate. Here, we focused on the two most diverse and ecologically important evergreen oaks: *Quercus aquifolioides* and *Quercus spinosa* in Qinghai–Tibetan Plateau (QTP), which is considered as world’s biodiversity hotspot. We genotyped 1,657 individuals of 106 populations at 15 nuclear microsatellite loci throughout the species distribution range. Spatial patterns of genetic diversity were identified by mapping the allelic richness (AR) and locally common alleles (LCA) according to the circular neighborhood methodology. Migration routes from QTP were detected by historical gene flow estimation. The response pattern of genetic variation to environmental gradient was assessed by the genotype–environment association (GEA) analysis. The overall genetic structure showed a high level of intra-species genetic divergence of a strong west-east pattern. The West-to-East migration route indicated the complex demographic history of two oak species. We found evidence of isolation by the environment in *Q. aqu*-East and *Q. spi*-West lineage but not in *Q. aqu*-West and *Q. spi*-East lineage. Furthermore, priority for conservation should be given to populations that retain higher spatial genetic diversity or isolated at the edge of the distribution range. Our findings indicate that knowledge of spatial diversity and migration route can provide valuable information for the conservation of existing populations. This study provides an important guide for species conservation for two oak species by the integration of population and landscape genetic methods.

## Introduction

The Qinghai–Tibet Plateau (QTP) is the highest and largest plateau with its southern (Himalayas) and southeastern border (Hengduan Mountains, HDM) considered as world’s biodiversity hotspots (Myers et al., 2000; Zhang et al., 2002; Mulch and Chamberlain, 2006; Wen et al., 2014). This plateau harbors abundant species richness with more than 12,000 species of vascular plants, many of which are alpine endemics (Wu et al., 1995; Liu et al., 2000). However, due to anthropogenic habitat loss or fragmentation and climate change, the species diversity has decreased rapidly and led to a sharp decrease in the natural distribution of some species in this region (Xu et al., 2017; Song et al., 2018). Hence, facing the crisis of diversity decrease in the QTP, establishing biodiversity richness areas of conservation priorities is considered one of the most effective strategies for halting the loss of biodiversity (Myers et al., 2000; Geldmann et al., 2018).

Population genetics approach is a useful tool for biodiversity conservation by detecting population substructure, measuring genetic diversity, and identifying potential risks associated with demographic change and inbreeding (Frankham, 1995). One limitation of this approach is the inability to assess spatial patterns of genetic diversity of species across species distribution ranges (Petit et al., 1998). The development of molecular tools in combination with population genetics and geographic information system (GIS) provides opportunities to carry out spatial analyses of genetic diversity patterns (Degen and Scholz, 1998). For example, allelic richness (AR) and locally common alleles (LCA) between circular neighborhoods of sampled populations can be used to interpolate genetic parameters (Hanotte et al., 2002; Hoffmann et al., 2003; Van Zonneveld et al., 2012). Furthermore, recently appearing landscape genetics or genomics approaches integrating genetic variations and landscape characteristics provide novel insights into the molecular basis of local adaptation and conservation strategies (e.g., Manel et al., 2010; Sork et al., 2013; McKinney et al., 2017; Feng and Du, 2022). Therefore, a combination of population and landscape genetics or genomics is likely to provide the best understanding of the molecular imprint of local adaptation and further guide the conservation strategies.

In addition to local adaptation, migrating to new favorable locations is also a response pattern of plants to rapid climate changes, which is important for species conservation (Wulff, 1943; Ozenda, 1988; Donoghue et al., 2001; Donoghue and Smith, 2004). Studies have suggested that plants from the QTP might undergo specific migration patterns, that is, the out-of-QTP hypothesis (Wen et al., 2014, and references therein). Recent phylogenetic studies from various plants have provided evidence to support this hypothesis. For example, *Gentiana* L. diversified initially on the QTP, then dispersed to eastern China, Europe, and other areas (Favre et al., 2016). Similar patterns have been reported in Allium L. Li M. J. et al. (2021), Lagotis Gaertn. Li et al. (2014), Rhodiola L. Zhang et al. (2014), and Picea A. Dietrich Lockwood et al. (2013) (see summary in Table 1 and reference in Qiu et al., 2011; Liu et al., 2012).

**TABLE 1:** Summary of plant studies on the out-of-QTP hypothesis.

Genus/Species	Family	Sample range	Methods	Migration route from QTP	References
*Allium* spp.	Amaryllidaceae	Europe, Caucasus and southwest Asia	cpDNA, ITS	To Caucasus and Europe.	Li M. J. et al., 2021
*Gentiana* spp.	Gentianaceae	Global	cpDNA, ITS	To eastern China, Taiwan, Europe, North and South America, Australia and New Guinea.	Favre et al., 2016
*Lagotis* spp.	Plantaginaceae	Southwest China, northeastern Russia, Kazakhstan and India	cpDNA, ITS	To the central Asian highlands, followed by the northward migration into the arctic.	Li et al., 2014
*Rhodiola* spp.	Crassulaceae	QTP, north-east Asia, Europe and North America	cpDNA, ITS	To eastern Asia, central Asia, Europe and North America.	Zhang et al., 2014
*Picea* spp.	Pinaceae	Eastern North America, western North America and QTP	ITS	To western North America and another dispersal into Taiwan.	Lockwood et al., 2013
*Anaphalis* spp.	Asteraceae	Asia and North America	ITS, ETS	To the eastern Himalayas, eastern Asia, western Himalayas, North America, and southeast Asia.	Nie et al., 2013
*Leontopodium* spp.	Asteraceae	Europe, central and eastern Asia	AFLP	To Mongolian and central China.	Safer et al., 2011
*Leontopodium* spp.	Asteraceae	Europe, north and east Asia	ITS, ETS	To middle Asia and eastern Europe.	Blöch et al., 2010
*Kelloggia* spp.	Rubiaceae	Eastern Asia and western north America	cpDNA	To western North America.	Nie et al., 2005
*Sophora davidii*	Fabaceae	QTP, Southeast and northeast China	cpDNA, ITS	To the southeast and northeast China.	Fan et al., 2013
*Hippophae rhamnoides*	Elaeagnaceae	Eastern Asia and Europe	cpDNA, ITS	To central Asia, Asia Minor/Europe, northern China and the Mongolian plateau.	Jia et al., 2012
*Lepisorus clathratus*	Polypodiaceae	QTP and north-central China	cpDNA	To the north-central China northward into the Altai.	Wang et al., 2011

*cpDNA: chloroplast DNA, ITS: internal transcribed spacers, ETS: external transcribed spacers, AFLP: Amplified Fragments Length Polymorphism.*

*Quercus* L. is one of the most diverse and ecologically important tree genera in the QTP and adjacent areas (Huang et al., 1999; Denk et al., 2018). Among these oaks in QTP, two evergreen oak species, *Quercus aquifolioides* Rehd. et Wils. and *Quercus spinosa* David ex Franchet, belonging to a species complex of the genus *Quercus* of section *Ilex*, are the most widely distributed oak species across QTP, HDM, and Qinling Mountains (QM) (Huang et al., 1999). Similar to other oak species, the two species are characterized by monoecious, outcrossing features, wind pollination, and seed dispersal by animals and gravity (Huang et al., 1999; Du et al., 2017; Meng et al., 2017). They display different geographically intraspecific lineages: *Q. spinosa* was diverged into West and East lineages (Feng et al., 2016; Ju et al., 2019), while *Q. aquifolioides* was divided into Tibet and Hengduan Mountains–Western Sichuan Plateau (HDM-WSP) (Du et al., 2017). A recent study further suggested that climatic shift triggered a split of two oak species between the cold highlands and warm lowlands (Meng et al., 2017). In addition, studies using ecological niche models (ENMs) suggested that the two species are relatively stable (Feng et al., 2016; Du et al., 2017; Meng et al., 2017; Ju et al., 2019), but might endure contraction because of spatial constraints, such as land use/cover and human influence (Liao et al., 2021). All the above studies have yielded a substantial understanding of the evolutionary history, phylogeographic patterns, and potential distribution of *Q. aquifolioides* and *Q. spinosa*. However, there are few studies focusing on oak species conservation in this region, despite now they were increasingly threatened by climatic change and habitat fragments. Here, we genotyped 1,657 oak individuals from 106 populations collected across four major regions: QTP, HDM, QM, and warm lowlands in East China based on a dense range-wide sampling of the two species. We aimed to identify the priority areas of *Q. aquifolioides* and *Q. spinosa* for conservation by a combination of population and landscape genetic approaches by answering the following questions: (1) What is the spatial pattern of genetic diversity of two species? (2) What is the species migration route from QTP? and (3) How do the species respond to the environmental gradients?

## Materials and Methods

### Field Sampling, DNA Isolation, and Microsatellite Genotyping

**FIGURE 1:**
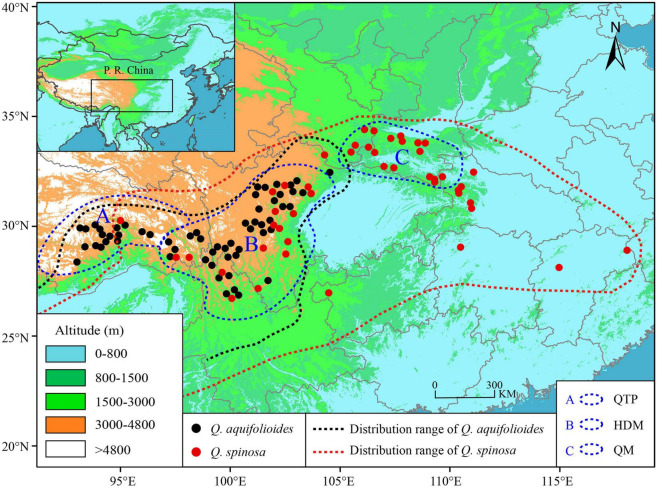
Geographic distribution and sampling sites of *Q. aquifolioides* and *Q. spinosa*. Black and red dashed lines indicate the geographic distribution of *Q. aquifolioides* and *Q. spinosa*, respectively. Three blue dashed lines represent defined research areas. The black rectangle on left top map represents the whole research area. QTP: Qinghai–Tibet Plateau, HDM: Hengduan Mountains, QM: Qinling Mountains.

We sampled leaf material from 996 individuals in 60 sites of *Quercus aquifolioides* and 661 individuals in 46 sites of *Quercus spinosa* throughout the species distribution range. The study sites were at least 30 km apart, and individuals were at least 100 m apart from each other to avoid sampling clone individuals. All leaf materials were rapidly dried in silica gel and stored for DNA isolation. The detailed information on sampling sites is depicted in Figure 1 and Supplementary Table 1.

Total genomic DNA was extracted from leaf samples for each individual using an improved cetyltrimethylammonium bromide (CTAB) method (Richards et al., 1994). We randomly selected one individual from each of six distant sites for pre-amplification experiments with 25 nuclear microsatellite (nSSR) loci developed for other oak species (Dow et al., 1995; Steinkellner et al., 1997; Kampfer et al., 1998; Ueno et al., 2008; Durand et al., 2010; Supplementary Table 2). We excluded loci harboring null alleles using MICRO-CHECKER 2.2.3 (Van Oosterhout et al., 2004). Departure from Hardy–Weinberg equilibrium (HWE) and linkage disequilibrium (LD) was evaluated using GenALEx 6 (Peakall and Smouse, 2006) and FDIST2 (Beaumont and Nichols, 1996). Finally, fifteen successfully amplified SSR loci were retained for subsequent analyses. The reaction procedures are modified from Du et al. (2017). The allele sizes were subsequently scored using GeneMarker v. 2.2 (Softgenetics, United States), and the genotypes were checked visually two times. A subset of the data, 959 individuals from 58 study sites of *Q. aquifolioides* at 15 nSSRs, were from Du et al. (2017) and Li Y. et al. (2021), and the additional data were first reported in this study.

### Genetic Diversity and Differentiation

We estimated genetic diversity indices including mean observed heterozygosity (*H*_*O*_), mean expected heterozygosity (*H*_*E*_), mean unbiased expected heterozygosity (*uH*_*E*_), mean effective population size (*N*_*E*_), and mean Shannon index (*I*) by GenAlEx 6 (Peakall and Smouse, 2006). The significance of genetic diversity was evaluated by *t*-test in SPSS 22 (SPSS Inc., Chicago, IL, United States) with a significance level of 0.05. In order to formulate optimal conservation strategies by revealing priority areas for conservation, we applied spatial analysis to improve the understanding of the geographic distribution of genetic diversity across the oak distribution range. We calculated and mapped the AR and LCA according to the circular neighborhood methodology described by Van Zonneveld et al. (2012). AR, also referred to mean number of alleles per locus, is a straightforward measure of genetic diversity based on molecular markers that aim at selecting populations for conservation (Frankel et al., 1995; Petit et al., 1998). LCA are alleles that occur in 25% or less of all grid cells and with a frequency of at least 5% in a grid cell per locus. Population with high LCA indicate the presence of genotypes adapted to specific environments; therefore, priority for conservation should be given to those populations (Frankel et al., 1995). After applying circular neighborhood to all samples, we calculated the AR and LCA for all 10-minute grid cells by GenAlEx 6 (Peakall and Smouse, 2006). AR was corrected by rarefaction to a minimum sample size of 10 re-sampled trees per cell with the HP-RARE software (Kalinowski, 2005).

We examined the genetic differentiation using hierarchical analysis of molecular variance (AMOVA, Excoffier et al., 1992) in Arlequin 3.5 (Excoffier and Lischer, 2010). The significance of fixation indices was tested using 10,000 permutations in Arlequin 3.5. We used a model-based clustering program implemented in STRUCTURE 2.3 (Pritchard et al., 2000) to infer the genetic clustering without consideration of sampling information. The program was run with the number of clusters (*K*) varied from 1 to 10 with 20 independent replicates conducted for each K-value, and the length of the burn-in period was set to 100,000 steps followed by the number of Markov chain Monte Carlo (MCMC) after burn-in of 100,000. We selected the optimal *K*-value by ΔK statistics performed in the web-based program STRUCTURE HARVESTER (Earl and Vonholdt, 2012). Graphic visualization of the STRUCTURE results was produced using DISTRUCT 1.1 (Rosenberg, 2004). We also conducted a principal component analysis (PCA) to visualize the genetic relatedness among individuals by calculating principal components (Novembre and Stephens, 2008) using “adegenet” R package (Jombart and Ahmed, 2011). The first two eigenvectors were plotted, and the discrete points reflect the real structure of populations. In addition, we conducted a principal coordinate analysis (PCoA, Gower, 1966) based on genetic covariance among populations in GenAlEx 6 (Peakall and Smouse, 2006) and plotted the first two eigenvectors to visualize genetic relatedness.

### Historical Gene Flow Among Lineages

The historical gene flow of two oak species was assessed by Migrate-n 3.6 (Beerli and Felsenstein, 2001; Beerli, 2006) based on the Bayes factor value. First, we generated initial θ (4*N*eμ, four times effective population size multiplied by mutation rate per site per generation) and *M* (immigration rate divided by the mutation rate) to estimate the amount and direction of gene flow. A continuous Brownian motion model and the default genetic differentiation were used to generate initial theta and migration values. Then, we started three independent MCMC chains with 500,000 iterations, respectively. We sampled every 100 steps under a constant mutation model and discarded the first 10,000 records as burn-in. After checking the model convergence, we calculated the mode value and 95% posterior probability.

### Genotype–Environment Associations

### Climatic Variables

We obtained climatic variables of the current conditions (∼1970–2000) from WorldClim^1^, a database of high spatial resolution global weather and climate data (Fick and Hijmans, 2017). A total of 31 climatic variables, including the full suite of 19 mean annual bioclimatic variables and 12 average monthly climate data for precipitation, were downloaded. We excluded climatic variables that were highly correlated with the threshold values of 0.7 using a variance inflation factor (VIF) test in “usdm” R package (Naimi et al., 2014). After avoiding the high multicollinearity bias, four climatic variables, namely precipitation seasonality (bio15, coefficient of variation), mean temperature of the driest quarter (bio09), temperature annual range (bio07, between the minimum temperature of the coldest month and the maximum temperature of the warmest month), and precipitation during June (prec06), were finally remained for downstream analyses (Supplementary Table 1).

### Linear Relationships

The linear relationships analysis can integrate environmental variables and spatial genetic structure into the analytical framework to assess the contributions of geography and environment in driving genetic differentiation (Feng and Du, 2022). The loading results of this analysis can be interpreted as the response proportion of environmental factors to genetic variation. In this study, we performed isolation-by-resistance (IBR) to illustrate the effects of the heterogeneous landscapes on the population genetic connectivity of two oak species in “ade4” R package (Dray and Dufour, 2007). We first predicted the potential distribution of two oak species based on the current ecological niche model (ENM) in MAXENT (Phillips and Dudik, 2008) and then transformed the environmental rasters into resistance surfaces. We generated the resistance distance based on circuit theory in CIRCUITSCAPE 4.0.5 (McRae, 2006; McRae et al., 2008) and “ResistanceGA” R package (Peterman, 2018). We performed Mantel tests of isolation by distance (IBD; Van Strien et al., 2015) and isolation by environment (IBE; Manthey and Moyle, 2015) to test the linear relationships between geographic or environmental distance and genetic distance using “ecodist” R package (Goslee and Urban, 2007). To distinguish the impact of IBD and IBE, a partial Mantel test was used to evaluate IBE/IBD by controlling the linear influence of geographic/environmental distance (Smouse et al., 1986). In addition, we performed multiple regression on distance matrices (MRM, Lichstein, 2007) to test the multivariate correlation between genetic distance matrix and climate distance using “ecodist” R package (Goslee and Urban, 2007). The significance for Mantel tests and MRM was evaluated by 10,000 permutation tests with the significance level set to 0.05.

We performed redundancy analyses (RDAs) to detect the multivariate relationship between genetic variation and climate variation (Van den Wollenberg, 1977; Legendre and Legendre, 1998) using “vegan” R package (Oksanen et al., 2017). A partial redundancy analysis (*p*RDAs, Legendre and Legendre, 1998, 2012) was performed to avoid the linear influence of geographic/climate variables when analyzing the climate/geographic variables. Statistical significance was evaluated from 999 permutations.

### Non-linear Relationships

A limitation of the linear relationships analysis is the inability to fit the variation in the rate of compositional turnover along environmental gradients and the curvilinear relationship between genetic distance and environmental and geographic distance. Therefore, a non-linear relationship is essential for applying the associated turnover function to each mapped environmental variable (Fitzpatrick and Keller, 2015). In this study, we performed generalized dissimilarity modeling (GDM) to identify non-linear relationships between genetic distance matrix (response variable) and geographic/environmental distances (predictors) using “gdm” package (Ferrier, 2002; Ferrier et al., 2007). We also evaluated the variation in the rate of allelic compositional change along environmental gradients by fitting splines (Fitzpatrick and Keller, 2015). Genetic distances among individuals were calculated based on allele frequency, and geographic distance was based on Euclidean distance among coordinates. We assessed the variable significance by randomization tests and assessed uncertainty due to sampling error by simulating 1,000 bootstrap iterations (Ferrier et al., 2007; Fitzpatrick et al., 2013).

## Results

### Genetic Diversity

We found that the genetic diversity was higher in *Q. aquifolioides* than in *Q. spinosa* (*H*_*O*_: 0.59 vs. 0.41; *H*_*E*_: 0.58 vs. 0.49; *uH*_*E*_: 0.61 vs. 0.52; *P* < 0.01) (Supplementary Table 3). We also identified significantly higher genetic diversity in *Q. aqu*-East than *Q. aqu*-West lineage (*H*_*O*_: 0.60 vs. 0.53; *H*_*E*_: 0.61 vs. 0.52; *uH*_*E*_: 0.64 vs. 0.54; *P* < 0.01) and a slightly higher genetic diversity in *Q. spi*-East lineage than *Q. spi*-West lineage (*H*_*O*_: 0.42 vs. 0.41; *H*_*E*_: 0.51 vs. 0.47; *uH*_*E*_: 0.53 vs. 0.50; *P* < 0.01) (Supplementary Table 3).

**FIGURE 2:**
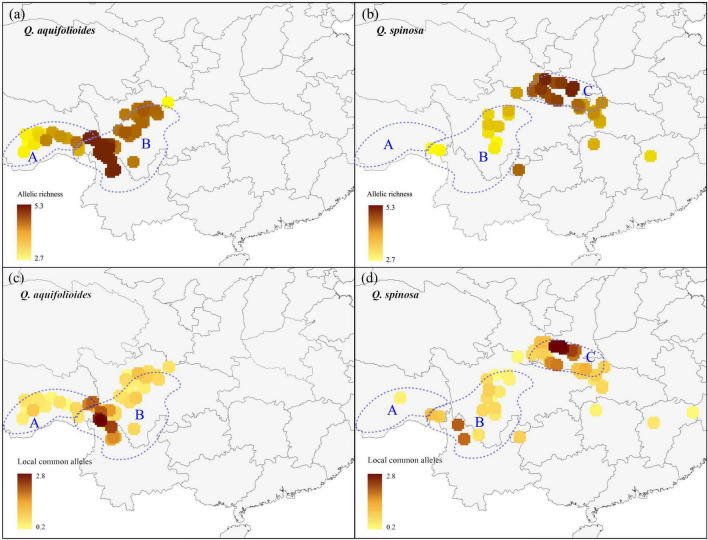
The allelic richness and locally common alleles map of *Q. aquifolioides* and *Q. spinosa.* The light blue dotted lines represent defined three research areas: **(a)** Qinghai–Tibet Plateau (QTP); **(b)** Hengduan Mountains (HDM); **(c)** Qinling Mountains (QM).

We applied a circular neighborhood re-sampling technique to ensure sufficiently and more evenly distributed data points for spatial diversity analysis. A total dataset of 31,872 trees for *Q. aquifolioides* and 21,152 trees for *Q. spinosa* was used for further AR and LCA analyses (Supplementary Figure 1). Our results showed that the enriched regions of AR and LCA of two oak species were different (Figure 2). For *Q. aquifolioides*, the populations located at HDM (*Q. aqu*-East lineage) in southwest Sichuan province and northwest Yunnan province contained higher AR and LCA than QTP (*Q. aqu*-West lineage) (Figures 2a,c). The marginal population PW of *Q. aquifolioides* located at the easternmost end of the HDM with a lower AR than in other areas of the HDM (Figure 2a). For *Q. spinosa*, populations from QM (*Q. spi*-East lineage) revealed higher AR and LCA than HDM (Figures 2b,d).

### Genetic Differentiation

**FIGURE 3:**
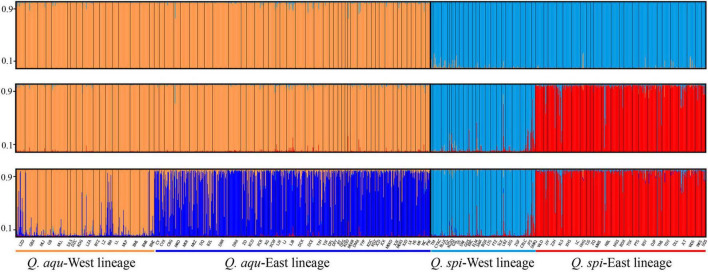
Individual assignment to two **(top)**, three **(middle)**, and four **(below)** genetic clusters by STRUCTURE of *Q. aquifolioides* and *Q. spinosa*. Each bar represents a single individual, with portions of the bar colored depending on the ancestry proportions estimated. The *y*-axis quantifies subgroup membership, and the *x*-axis shows the sample ID for each individual.

**FIGURE 4:**
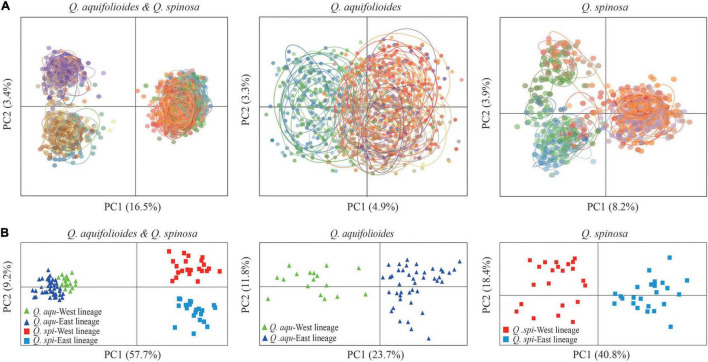
Genetic covariance of *Q. aquifolioides* and *Q. spinosa*. **(A)** Principal component analysis (PCA) plots based on genetic covariance among individuals. The first two principal components (PCs) are shown; **(B)** principal coordinate analysis (PCoA) plots of the first two components based on genetic covariance among populations.

Bayesian clustering identified *K* = 2 as the optimal number of evolutionary clusters (Supplementary Figure 2), subdivided all individuals into two clusters, one corresponded to *Q. aquifolioides* and the other to *Q. spinosa*. When *K* = 3, *Q. aquifolioides* was maintained unchanged while *Q. spinosa* is further subdivided into two geographically related lineages: *Q. spi*-West (22 sites from HDM) and *Q. spi*-East lineage (24 sites from QM and lowlands in East China). When *K* = 4, *Q. aquifolioides* is divided into *Q. aqu*-West (17 sites from QTP) and *Q. aqu*-East lineage (43 sites from HDM) (Figure 3). The results of PCA and PCoA were largely consistent with the STRUCTURE analysis with clear separation in interspecific and intraspecific levels (Figure 4). AMOVA showed a high level of genetic differentiation between *Q. aquifolioides* and *Q. spinosa*, and most of the variation occurred within populations (*F*_ST_ = 0.35, 65.4%) (Table 2).

**TABLE 2:** Hierarchical analyses of molecular variance (AMOVA) of *Q. aquifolioides* and *Q. spinosa* populations.

	d.f.^1^	SS^2^	VC^3^	Percentage of variation (%)	Fixation indices
**All samples**					
Between species	1	2738.131	1.70784	26.1	*F*_CT_ = 0.26
Among populations within species	104	2253.579	0.55798	8.5	*F*_SC_ = 0.12
Within populations	3208	13710.155	4.27383	65.4	*F*_ST_ = 0.35
***Q. aquifolioides***					
Between lineage	1	205.911	0.21585	4.4	*F*_CT_ = 0.04
Among populations within lineages	58	728.025	0.24428	4.9	*F*_SC_ = 0.05
Within populations	1932	8704.617	4.5055	90.7	*F*_ST_ = 0.09
***Q. aqu*-West lineage**					
Among populations	16	179.949	0.18603	4.4	*F*_ST_ = 0.04
Within populations	647	2621.183	4.05129	95.6	
***Q. aqu*-East lineage**					
Among populations	42	553.323	0.27335	5.4	*F*_ST_ = 0.05
Within populations	1285	6126.11	4.7674	94.6	
***Q. spinosa***					
Between lineages	1	324.207	0.48268	9.6	*F*_CT_ = 0.09
Among populations within lineages	44	983.166	0.64466	12.9	*F*_SC_ = 0.14
Within populations	1276	4963.162	3.88963	77.5	*F*_ST_ = 0.22
***Q. spi*-West lineage**					
Among populations	21	462.271	0.8055	18.1	*F*_ST_ = 0.18
Within populations	482	1767.044	3.66607	81.9	
***Q. spi*-East lineage**					
Among populations	23	520.894	0.54821	11.99	*F*_ST_ = 0.12
Within populations	794	3196.118	4.02534	88.01	

*Significance tests (1,000 permutations) showed all fixation indices were significant (P < 0.001). ^1^d.f., degrees of freedom; ^2^SS, sum of squares; ^3^VC, variance component.*

### Historical Gene Flow Among Lineages

The Migrate-n analysis generated θ and *M* values greater than zero, which revealed an asymmetric historical gene flow between two species, mainly from *Q. aquifolioides* to *Q. spinosa* (56.1 vs. 47.5) (Table 3). Moreover, we found gene movements occurred predominantly from *Q. aqu*-West into *Q. aqu*-East lineage (46.6 vs. 34.5) and from *Q. spi*-West into *Q. spi*-East lineage (65.6 vs. 43.0) (Table 3).

**TABLE 3:** Historical gene flow as estimated by Migrate-n among *Q. aquifolioides* and *Q. spinosa* based on SSR datasets.

		*N*_*e*_ *m*					
	θ	*Q. aquifolioides* →	*Q. aqu*-West →	*Q. aqu*-East →	*Q. spinosa →*	*Q. spi*-West →	*Q. spi*-East →
							
*Q*. *aquifolioides*	2.0 [1.5-2.3]				47.5 [34.3-58.1]		
*Q. aqu*-West	4.0 [3.3-4.6]			34.5 [31.7-36.6]		30.6	10.2
						[24.7-35.9]	[5.5-15.1]
*Q. aqu*-East	7.7 [7.2-8.1]		46.6 [43.7-49.1]			36.6 [31.7-41.5]	17.9 [10.1-24.1]
*Q*. *spinosa*	3.5 [2.4-4.5]	56.1 [33.6-49.1]					
*Q. spi*-West	8.4 [7.2-8.8]		34.7	56.8			43
			[24.5-44.1]	[43.7-69.1]			[39.3-46.3]
*Q. spi*-East	2.0 [1.5-2.3]		27.3	26.6		65.6	
			[19.1-35.4]	[13.7-39.1]		[52.9-69.6]	

*The values in square brackets give the 95% credibility interval; θ, 4Neμ; →, source populations; Ne, effective population size; M, mutation-scaled immigration rate; m, immigration rate; μ, mutation rate.*

### Linear Relationships

**FIGURE 5:**
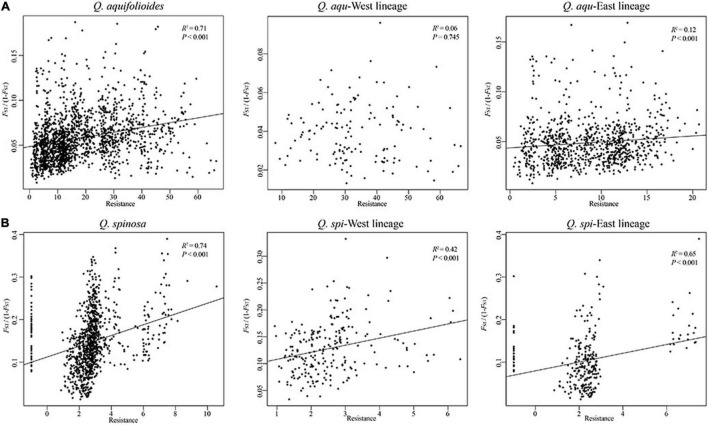
Relationship of genetic distance (*F*_*ST*/_(1-*F*_*ST*_)) and resistance distance based on climatic niche suitability of **(A)** *Q. aquifolioides* and **(B)** *Q. spinosa*.

Our analyses revealed highly significant correlations between pairwise genetic distances and resistance distance in *Q. aquifolioides* and *Q. spinosa*, but not in *Q. aqu-*West lineage (*P* = 0.745). The pattern of IBR in *Q. spi*-East was stronger than in *Q. spi*-West lineage (*R*^2^ = 0.65 vs. *R*^2^ = 0.42; Figure 5). In addition, we found significant patterns of IBD and IBE by Mantel and partial Mantel tests in *Q. aquifolioides* and *Q. spinosa* (Table 4 and Supplementary Figure 3). However, significant IBD was only detected in *Q. aqu-*West and *Q. spi*-East lineage; significant IBE was detected in *Q. aqu-*East and *Q. spi*-West lineage (Table 4 and Supplementary Figure 3). More specifically, genetic distance was significantly associated with annual range temperature (bio07) and seasonal precipitation (bio15) in *Q. aquifolioides* and *Q. aqu*-East lineage; bio15 and precipitation during June (prec06) in *Q. spinosa* and *Q. spi*-West lineage (Supplementary Table 4). These findings were consistent with optimal MRM models that yielded similar results (Supplementary Table 5).

**TABLE 4:** Mantel tests and partial Mantel tests (conditioned with geographic or environmental distances) between pairwise genetic distance (*F*_*ST*_/(1 - *F*_*ST*_)) and geographic or environmental distances in different lineages and all populations of *Q. aquifolioides* and *Q. spinosa*.

	*Q. aquifolioides*		*Q. aqu*-West lineage		*Q. aqu*-East lineage		*Q. spinosa*		*Q. spi*-West lineage		*Q. spi*-East lineage	
	Mantel’s *r*	*P*	Mantel’s *r*	*P*	Mantel’s *r*	*P*	Mantel’s *r*	*P*	Mantel’s *r*	*P*	Mantel’s *r*	*P*
***Mantel test***												
Isolation by Distance (IBD)	0.52	** < 0.001**	0.15	**0.006**	0.39	** < 0.001**	0.55	** < 0.001**	0.38	** < 0.001**	0.49	**0.005**
Isolation by Environment (IBE)	0.18	**0.025**	–0.07	0.675	0.3	**0.014**	0.28	** < 0.001**	0.25	**0.006**	0.44	0.005
***partial Mantel test***												
IBD conditioned with environmental distances	0.47	** < 0.001**	0.24	**0.007**	0.31	0.059	0.5	** < 0.001**	0.13	0.052	0.25	**0.044**
IBE conditioned with geographical distances	0.21	**0.031**	–0.2	0.875	0.19	** < 0.001**	0.01	**0.048**	0.32	**0.002**	–0.02	0.566

*The bolded text indicates that data are significant.*

The percentages of variance explained by RDA and *p*RDA were similar, and we thus report results for *p*RDA. Geography (4.0 and 5.2%) explained more genetic variance than climate variables (1.5 and 3.0%) in *Q. aqu*-West and *Q. spi*-East lineage, whereas climate variables (2.7 and 5.6%) explained more genetic variance than geography (1.4 and 5.1%) in *Q. aqu*-East and *Q. spi*-West lineage (Table 5). Partitioning of the total genetic variance revealed that bio07 and prec06 explained most genetic variance in *Q. aqu*-East lineage (44.5 and 23.5%), while in *Q. spi*-West lineage, prec06 and bio15 were the two most explanatory environmental variables (46.0% and 20.3%) (Table 5 and Supplementary Figure 4).

**TABLE 5:** Summary of the genetic variations associated with climate and geographic variables based on RDA and *p*RDA in *Q. aquifolioides* and *Q. spinosa*.

	RDA			*p*RDA				RDA			*p*RDA		
	PVE	Eigenvalue	*P*	PVE	Eigenvalue	*P*		PVE	Eigenvalue	*P*	PVE	Eigenvalue	*P*
***Q. aquifolioides***							***Q. spinosa***						

climate	3.3	8.46	0.001	1.44	4.9	0.001	climate	10.34	18.91	0.001	2.67	5.1	0.001
geography				1.89	7.47	0.001	geography				4.13	15.79	0.001
bio15	33.51	11.33	0.001	15.19	2.97	0.002	bio15	60.79	45.99	0.001	27.11	5.53	0.001
bio09	37.06	12.54	0.001	50.62	3.92	0.001	bio09	17.14	12.96	0.001	25.11	5.12	0.001
bio07	14.28	4.83	0.001	11.69	2.29	0.004	bio07	14.14	10.69	0.001	23.72	4.83	0.001
prec06	15.15	5.12	0.001	22.5	4.41	0.001	prec06	7.93	5.99	0.001	24.06	4.91	0.001
Whole model			0.001			0.001	Whole model			0.001			0.001

***Q. aqu*-West lineage**							***Q. spi*-West lineage**						

climate	3.16	2.67	0.001	1.45	3.43	0.001	climate	7.58	5.07	0.001	5.62	3.94	0.001
geography				4.03	2.47	0.001	geography				5.12	7.18	0.001
bio15	18.08	1.93	0.029	23.78	3.26	0.002	bio15	25.67	3.2	0.002	20.31	3.2	0.001
bio09	30.83	3.29	0.001	30.76	4.22	0.001	bio09	23.41	4.74	0.001	16.87	2.65	0.003
bio07	40.88	4.37	0.001	29.41	4.03	0.001	bio07	15.76	5.2	0.001	10.78	2.66	0.004
prec06	10.21	1.09	0.354	16.05	2.2	0.001	prec06	35.16	7.12	0.001	46.04	7.26	0.001
Whole model			0.001			0.001	Whole model			0.001			0.001

***Q. aqu*-East lineage**							***Q. spi*-East lineage**						

climate	3.66	6.25	0.001	2.7	4.66	0.001	climate	6.04	6.48	0.001	2.98	5.74	0.001
geography				1.38	4.75	0.001	geography				5.19	6.57	0.001
bio15	27.17	6.79	0.001	17.27	2.73	0.001	bio15	32.37	8.4	0.001	19.2	4.4	0.001
bio09	14.53	9.3	0.001	14.64	8.3	0.001	bio09	30.59	7.94	0.001	26	5.97	0.001
bio07	37.19	3.63	0.001	44.57	3.22	0.001	bio07	11.28	2.93	0.001	16.4	3.76	0.001
prec06	21.11	5.28	0.001	23.52	4.38	0.001	prec06	25.76	6.69	0.001	38.4	8.81	0.001
Whole model			0.001			0.001	Whole model			0.001			0.001

*PVE, percentage of explained variance.*

### Non-linear Relationships

**FIGURE 6:**
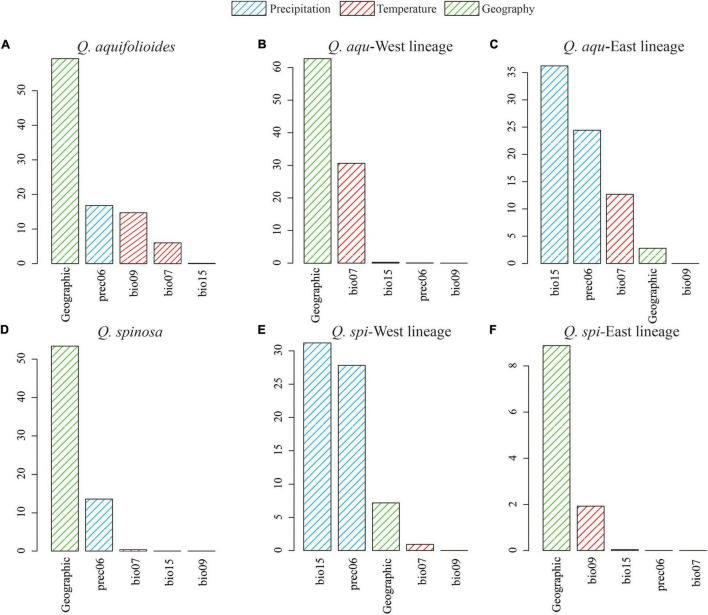
Variable importance of environmental variables based on analysis of generalized dissimilarity model (GDM) for **(A)** all populations of *Q. aquifolioides*, **(B)** *Q. aqu*-West lineage, **(C)** *Q. aqu*-East lineage, **(D)** all populations of *Q. spinosa*, **(E)** *Q. spi*-West lineage, and **(F)** *Q. spi*-East lineage.

Generalized dissimilarity modeling analyses suggested that geography was the most important predictor among all variables considered in *Q. aquifolioides*, *Q. aqu*-West, *Q. spinosa*, and *Q. spi*-East lineage (59.3, 62.9, 53.4, and 8.8%) (Supplementary Table 6). However, there was almost no contribution of geography in *Q. aqu*-East and *Q. spi*-West lineage (2.8 and 7.2%), while bio15 was the most important environmental factor (36.2 and 30.8%) (Figure 6 and Supplementary Table 6). These results were consistent with I-spline analysis (Supplementary Figure 5).

## Discussion

Population and landscape genetic methods were used to identify priority areas for conservation throughout the species range of *Q. aquifolioides* and *Q. spinosa*. We found that the two evergreen oak species might originate from QTP and then dispersal into HDM and QM. In addition, the intraspecific genetic variation of different lineages of the two species showed different response patterns to environmental factors. Therefore, priority conservation areas were different for the two species: for *Q. aquifolioides*, a priority area for conservation should be at HDM, whereas for *Q. spinosa*, populations from QM should be considered in conservation.

### The West-To-East Migration Route From Qinghai–Tibetan Plateau

*Quercus aquifolioides* and *Quercus spinosa* were assigned to two distinct genetic clusters (Figures 3, 4), and the direction of the interspecific and intraspecific gene flow was from west to east (Table 3). These results showed a strong West-to-East migration pattern and likely reflect long-term geographic isolation due to the orogenic history of QTP and adjacent regions (Qiu et al., 2011; Wen et al., 2014). In addition, the amount of historical gene flow was asymmetric, mainly from *Q. aquifolioides* to *Q. spinosa* at the interspecific level (56.1 vs. 47.5) and West lineages to East lineages at the intraspecific level (46.6 vs. 34.5 and 65.6 vs. 43.0) indicating the asymmetric introgression of the species (Currat et al., 2008; Du et al., 2011).

There are already several migration routes for the out-of-QTP hypothesis (Table 1), and all of the studies suggested that migration, orographic, and climate oscillations catalyzed intraspecific differentiation, diversification, and adaptation of species in this region (see the summary of Wen et al., 2014). It is suggested that orographic and climatic oscillations might result in lots of small fragmented habitats with different microclimates, which could influence the direction of natural selection, and might promote intraspecific high differentiation of species (Sobel et al., 2010). Our results represent a very typical case of a West-to-East migration pattern, which might be triggered by extensive uplifts of the QTP (see the summary of Favre et al., 2015). The QTP uplift events provided opportunities for the ancestral population in this region continually expanded to its eastward ranges and gradually triggered and facilitated speciation and diversifications of oak species (Zhou, 1992). Meanwhile, the West-to-East migration pattern indicated that migrating to new favorable locations might be a survival strategy of species to rapid climate changes as in *Sophora davidii* (Fan et al., 2013) and *Gentiana* (Favre et al., 2016).

### Response Pattern of Genetic Variation Under Genotype–Environment Association

Genotype–environment association (GEA) analysis, including mantel tests, redundancy analyses, and generalized dissimilarity modeling, integrates environmental variables and spatial genetic structure into the analytical framework to detect the adaptive variation (Feng and Du, 2022). GEA analysis is essential for understanding the mechanisms underlying the evolutionary responses to environments and was used to quantify patterns of interaction between genetic variation and climate conditions (Hansen et al., 2012). In addition, resistance analysis is important to understand how the species respond to different resistance distances (McRae, 2006). Based on this theory, we identified the landscape resistance matrix that was most highly correlated with pairwise genetic distances in *Q. aquifolioides* and *Q. spinosa*, especially in *Q. aqu*-East and *Q. spi*-East lineage (Figure 5), and this result may be related to increased habitat isolation in this area resulted from the disjunct distribution of the two oak species. However, the pairwise genetic distance was not correlated with resistance distance in *Q. aqu*-West lineage (Figure 5), indicating that populations from this lineage are low resistant to dispersal and might endure high genetic connectivity among populations. This result is confirmed with the lowest genetic differentiation in the *Q. aqu*-West lineage (Table 2).

The IBD and IBE results indicated the intraspecific lineages of oaks with different response patterns of genetic variation. We detected significant IBD patterns in *Q. aqu*-West and *Q. spi*-East lineage (Table 4 and Supplementary Figure 3, Supplementary Table 4). These results were consistent with GDM and RDA (Figure 6, Table 5 and Supplementary Figure 4, Supplementary Table 6), indicating that the genetic variation of *Q. aqu*-West and *Q. spi*-East lineage was mainly driven by selectively neutral evolutionary processes, not by strong selection pressure from the environment. The complex geological structure of mountains might form a natural geographic barrier for seeds or pollen dispersal, which can provide potential conditions for the formation and independent evolution of plants’ intraspecific lineages (Liu et al., 2013; Li et al., 2014).

By contrast, we detected significant IBE patterns in *Q. aqu*-East and *Q. spi*-West lineage (Table 4 and Supplementary Figure 5, Supplementary Tables 5, 6), where the extreme environmental conditions on the plateau might be regarded as a significant climatic barrier, rather than a geographic barrier. It also suggests that geographic isolation may cause interspecific and intraspecific differentiation; adaptation to local climate and environmental factors reinforces this differentiation and gradually forms this significant IBE pattern (Gao et al., 2021). Accordingly, GDM and RDA both suggesting temperature annual range (bio07) and precipitation during June (prec06) were the most significant environmental factor driving genetic variation in *Q. aqu*-East and *Q. spi*-West lineage (Figure 6, Table 5 and Supplementary Figure 4, Supplementary Table 6), respectively. The temperature may be the main driver of genetic variation for *Q. aqu*-East lineage, and it may influence the growth of plants in microhabitats by affecting the metabolic processes (Wahid et al., 2007). Precipitation might have a great impact on phenological and growth of oak from Q. spi-West lineage and then affect the ability to adapt to climate change.

### Priority Areas for Conservation

A better understanding of the spatial distribution of genetic diversity is necessary for the formulation of effective and efficient conservation strategies (Petit et al., 1998). Priority for conservation should be given to populations that retain the highest AR and LCA because the likelihood to find interesting breeding materials is higher in the highest genetic diversity populations, which can indicate the presence of genotypes adapted to specific environments (Frankel et al., 1995; Tanksley and McCouch, 1997). We found that the priority conservation areas were different for the two species based on a large number of samples (1,657 individuals) across their distribution range (Figure 2). For *Q. aquifolioides*, a priority area for conservation should be the populations located at HDM (*Q. aqu*-East lineage), which contained the highest AR and LCA. In addition, the marginal population PW of *Q. aquifolioides* located at the easternmost end of the HDM with a lower AR and LCA than other areas. Risk of non-adaptedness (RONA) revealed that this marginal population might be at higher risk of extinction under future climate (Du et al., 2020). Therefore, populations isolated at the edge of the distribution range also should be considered in conservation activities to prevent the extinction of species in this area. For *Q. spinosa*, a priority area for conservation should be the populations located at QM (*Q. spi*-East lineage) with the highest genetic diversity. The second area of a higher diversity of *Q. spinosa* is located on the border zone between Sichuan and Yunnan province, probably related to the high species richness of this region. The higher AR and LCA in the *Q. aqu*-East and *Q. spi*-East lineage than in *Q. aqu*-West and *Q. spi*-West lineage suggested those populations received more immigrants and played an important role in evolution and diversification (López-Pujol et al., 2011; Favre et al., 2015), while the lower genetic diversity in *Q. aqu*-West and *Q. spi*-West lineage likely reflects population contraction or extinction–recolonization dynamics in this area (Qiu et al., 2011; Du et al., 2017; Meng et al., 2017).

## Data Availability Statement

The original contributions presented in the study are included in the article/Supplementary Material, further inquiries can be directed to the corresponding author/s.

## Author Contributions

FD designed the research. KL performed the experiments and the analyses. KL, MQ, and FD wrote the manuscript. All authors contributed to its revision.

## Conflict of Interest

The authors declare that the research was conducted in the absence of any commercial or financial relationships that could be construed as a potential conflict of interest.

## Publisher’s Note

All claims expressed in this article are solely those of the authors and do not necessarily represent those of their affiliated organizations, or those of the publisher, the editors and the reviewers. Any product that may be evaluated in this article, or claim that may be made by its manufacturer, is not guaranteed or endorsed by the publisher.

## Footnotes

1
http://www.worldclim.org/version2
