# Supplementary figures and images for: Population and Landscape Genetics Provide Insights Into Species Conservation of Two Evergreen Oaks in Qinghai–Tibet Plateau and Adjacent Regions
Source: Front Plant Sci. 2022 May 19;13:858526. doi: 10.3389/fpls.2022.858526 (PMC9161217; doi:10.3389/fpls.2022.858526)

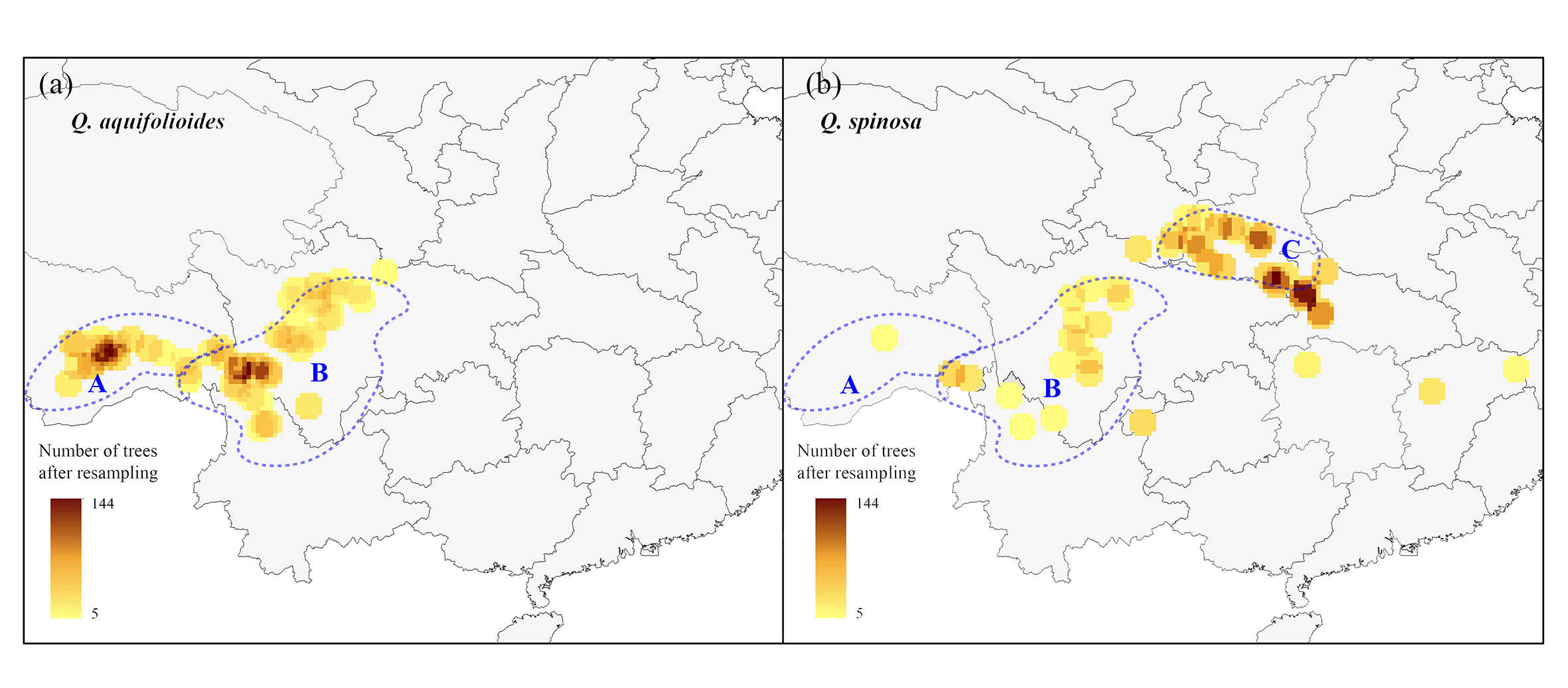

Supplement: Supplementary file 1 [file Image_1.jpg]

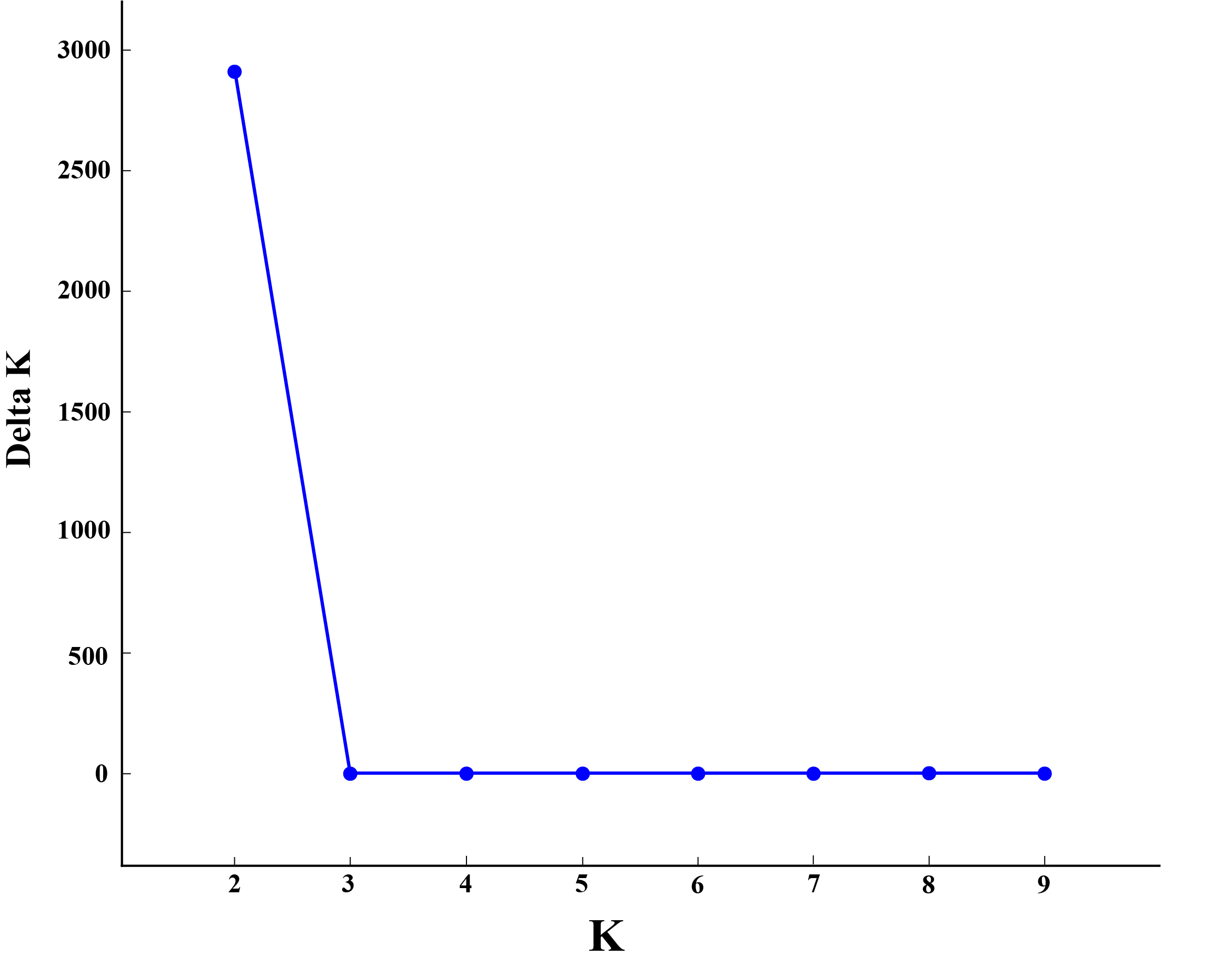

Supplement: Supplementary file 2 [file Image_2.JPEG]

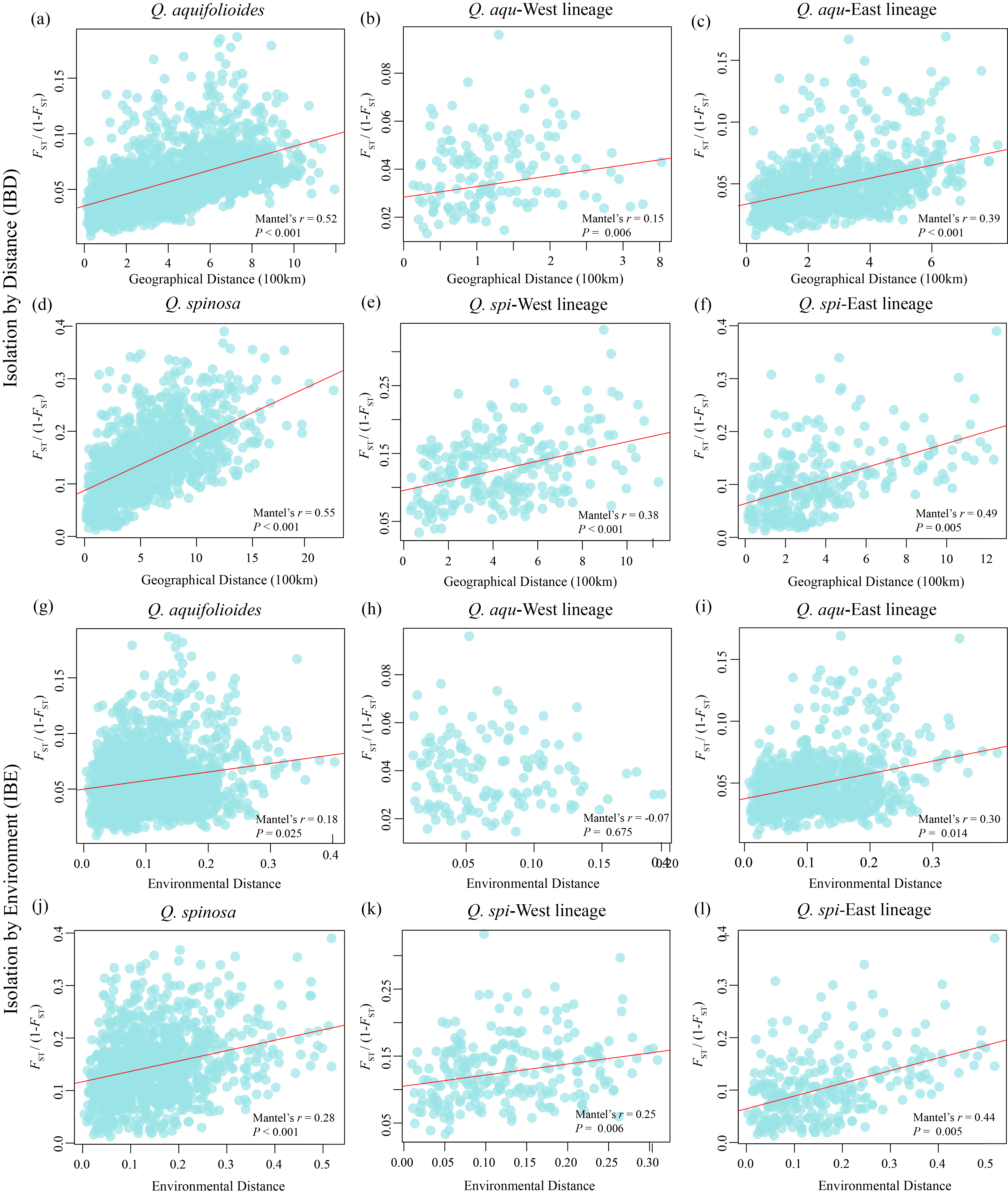

Supplement: Supplementary file 3 [file Image_3.JPEG]

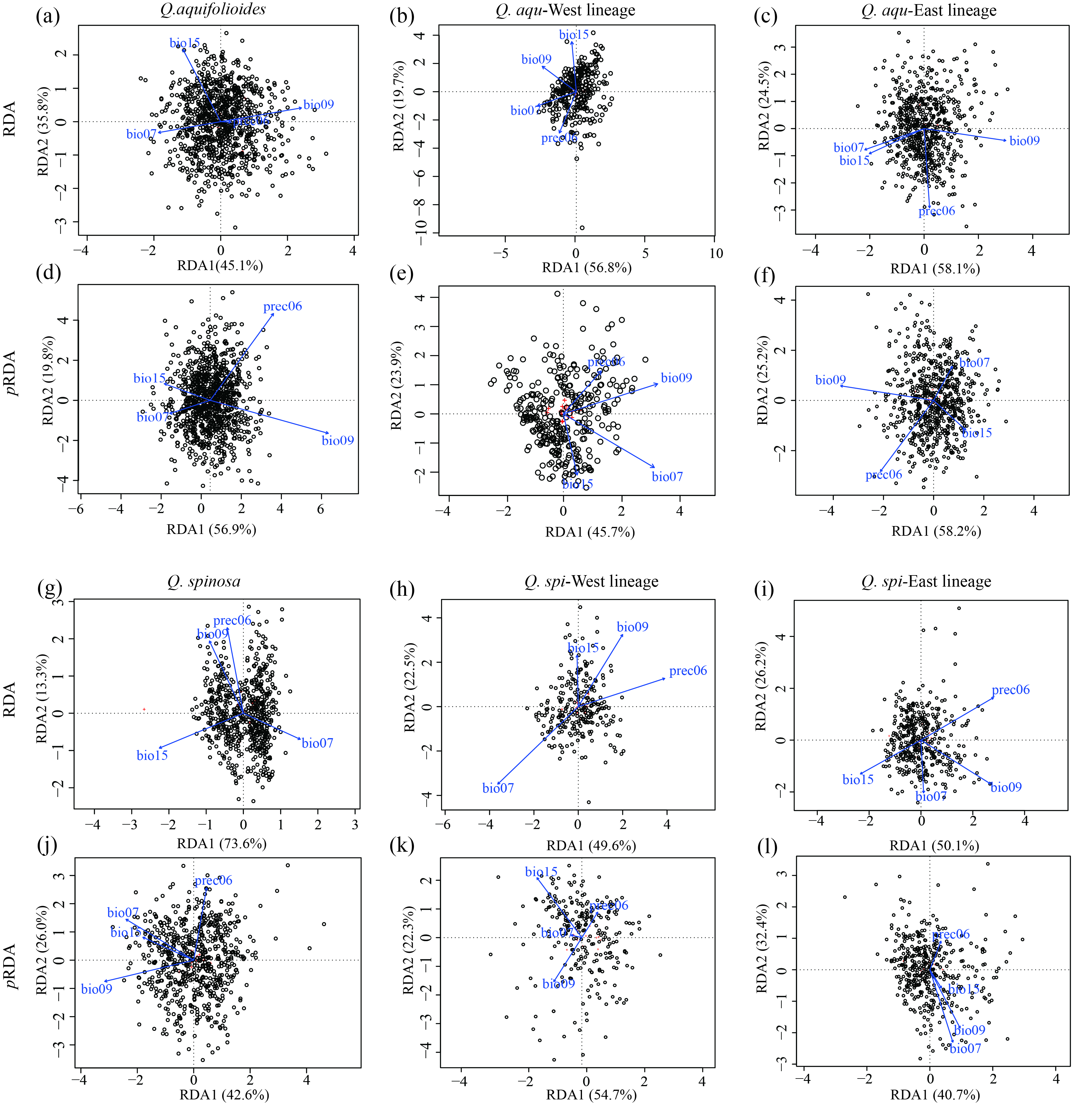

Supplement: Supplementary file 4 [file Image_4.JPEG]

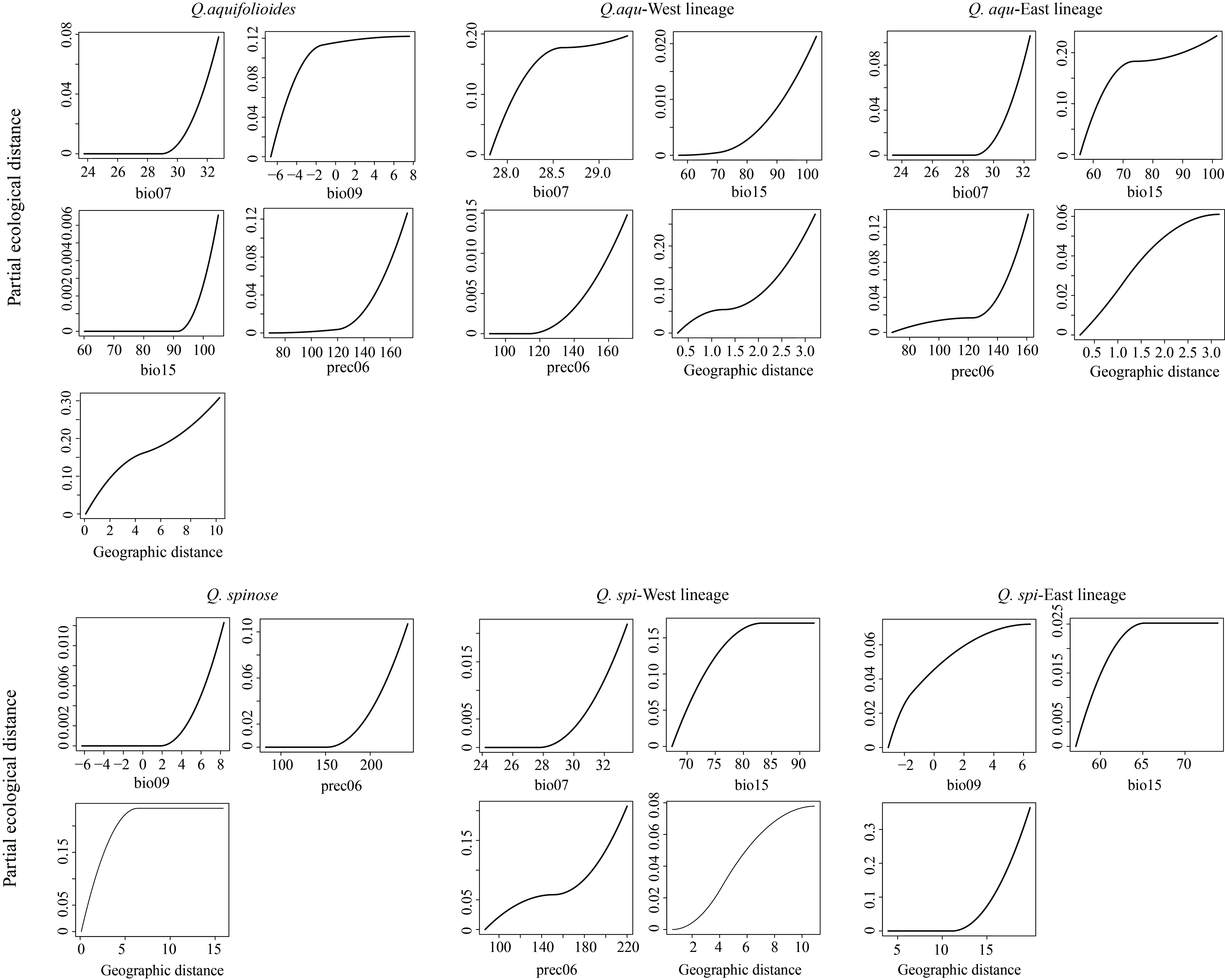

Supplement: Supplementary file 5 [file Image_5.JPEG]
