# Supplementary material for: Population and Landscape Genetics Provide Insights Into Species Conservation of Two Evergreen Oaks in Qinghai–Tibet Plateau and Adjacent Regions
Source: Front Plant Sci. 2022 May 19;13:858526. doi: 10.3389/fpls.2022.858526 (PMC9161217; doi:10.3389/fpls.2022.858526)
Supplement: Supplementary file 6 [file Data_Sheet_1.docx]

**SUPPORTING MATERIAL**

**Figure S1.** The map of number of trees per grid after resampling of *Q.aquifolioides* and *Q. spinosa* were made with a 10-minutes grid applying a one-degree circular neighborhood.

**Figure S2**. Variation of ∆K calculated as ∆K = m|L′′(K) | / s[L(K)]. The modal value of this distribution is the true K(*) or the uppermost level of structure..

**Figure S3.** Mantel tests of genetic distance (*F*_ST_ / (1 - *F*_ST_)) against geographic and environmental distances of *Q. aquifolioides* and *Q. spinosa*. Regressions of isolation by distance between genetic distance and geographic distance among (a) all populations of *Q. aquifolioides*, (b) *Q. aqu*-West lineage, (c) *Q. aqu*-East lineage, (d) all populations of *Q. spinosa*, (e) *Q. spi*-West lineage, (f) *Q. spi*-East lineage. Regressions of isolation by environment between genetic distance and environmental distance among (g) all populations of *Q. aquifolioides*, (h) *Q. aqu*-West lineage, (i) *Q. aqu*-East lineage, (j) all populations of *Q. spinosa*, (k) *Q. spi*-West lineage and (l) *Q. spi*-East lineage. Red lines show how the estimated genetic distance changes between plot pairs.

**Figure S4.** Triplots of redundancy analysis (RDA) and partial redundancy analysis (*p*RDA) results for *Q.aquifolioides* (upper panels) and *Q. spinosa* (lower panels). The plots show the first and second constrained axes from RDA. Environmental factors are depicted as black vectors (arrows), where the length of the vector is a representation of the magnitude of the contribution of that environmental variable in explaining variance. The angle between environmental variable vectors is a representation of the correlation between those variables.

**Figure S5.** Cumulative importance of genetic variation along environmental gradients generalized dissimilarity model (GDM) in each lineage and all populations of *Q.aquifolioides* and *Q. spinosa*. I-spline plot which coefficients equal zero is not shown.

**Table S1.** The geographic, climate and sample informations of sample sites of *Q.aquifolioides* and *Q. spinosa* used in the study. Code, sampling population name; N, sample sizes; bio15, precipitation seasonality (coefficient of variation); bio09, mean temperature of driest quarter; bio07, temperature annual range; prec06, precipitation during June.

| **Code** | **Collection site** | **Longitude(E)** | **Latitude(N)** | **N** | **bio15** | **bio09** | **bio07** | **prec06** |
| --- | --- | --- | --- | --- | --- | --- | --- | --- |
| ***Quercus aquifolioides*** | | | | | | | | |
| LZD | Douyu, Longzi, Tibet | 93.02 | 28.37 | 21 | 99.36 | 0.77 | 27.6 | 97 |
| GBX | Jiangda, Gongbujiangda, Tibet | 93.16 | 29.92 | 30 | 105.04 | -2.47 | 29.2 | 93 |
| MLJ | Jjiage, Milin, Tibet | 93.38 | 29.07 | 20 | 103.3 | 1.53 | 27.7 | 90 |
| GB | Gongbujiangda, Tibet | 93.42 | 29.88 | 13 | 102.48 | -1.35 | 27.7 | 98 |
| MLL | Lilong, Milin, Tibet | 93.87 | 29.12 | 39 | 95.98 | 2.37 | 29.4 | 112 |
| SJLS | Sejila Mts., Tibet | 93.98 | 29.18 | 7 | 94.58 | 1.63 | 28.2 | 115 |
| DZC | Dazhao, Tibet | 94.12 | 29.97 | 14 | 92.58 | -5.22 | 28.4 | 113 |
| KDG | Kadinggou, Tibet | 94.15 | 29.73 | 15 | 94.04 | -1.18 | 27 | 110 |
| LZA | Linzhi airport , Milin, Tibet | 94.34 | 29.3 | 26 | 91.27 | 2.17 | 28 | 127 |
| BYZ | Bayi Town, Tibet | 94.35 | 29.67 | 15 | 91.97 | 1.33 | 27.8 | 123 |
| LZ | Linzhi, Tibet | 94.52 | 29.55 | 15 | 90.18 | -3.42 | 27.7 | 115 |
| BM | Bomi, Tibet | 94.72 | 29.64 | 16 | 89.75 | -6.67 | 28.7 | 116 |
| LL | Lulang, Tibet | 94.77 | 29.85 | 15 | 90.94 | -3.95 | 28.8 | 126 |
| MLP | Pai Town, Milin, Tibet | 94.83 | 29.46 | 28 | 89.7 | -1.22 | 28 | 136 |
| BMS | Suotong, Bomi, Tibet | 95.21 | 30.06 | 23 | 91.03 | 3.4 | 28.2 | 173 |
| BMR | Ranwu, Bomi, Tibet | 95.99 | 29.75 | 23 | 88.84 | -1.1 | 28.5 | 147 |
| BMZ | Zhongba, Bomi, Tibet | 96.35 | 29.62 | 12 | 86.95 | -3.88 | 29.1 | 127 |
| CY | Guyu, Chayu, Tibet | 97.19 | 29.29 | 12 | 82.45 | -4.65 | 29.4 | 122 |
| CYX | Xiongjiu, Chayu, Tibet | 97.32 | 28.61 | 13 | 88.77 | 5.42 | 27.6 | 256 |
| CBG | Cibagou, Chayu, Tibet | 97.46 | 28.89 | 22 | 84.66 | 1.95 | 26.4 | 174 |
| MKD | Dengba, Mangkang, Tibet | 98.18 | 29.57 | 16 | 85.16 | -8 | 29.1 | 93 |
| MKR | Rumei, Mangkang, Tibet | 98.47 | 29.72 | 22 | 88.11 | -4.9 | 30.9 | 91 |
| MKZ | Zixu, Mangkang, Tibet | 98.65 | 29.43 | 19 | 83.34 | -3.65 | 30.1 | 88 |
| DQ | Deqing, Yunnan | 98.96 | 28.44 | 17 | 68.21 | -3.03 | 27.5 | 68 |
| BZL | Benzilan, Yunnan | 99.15 | 28.31 | 20 | 68.87 | -1.43 | 25.6 | 77 |
| DRR | Rilong, Derong, Sichuan | 99.23 | 28.86 | 38 | 78.43 | -3.15 | 26.1 | 84 |
| DRX | Derong-Xiangcheng, Sichuan | 99.41 | 29.06 | 30 | 84.57 | 0.2 | 31.4 | 95 |
| ZD | Zhongdian, Yunnan | 99.64 | 27.9 | 13 | 67.38 | -0.47 | 27.8 | 85 |
| XCD | Xiangcheng-Derong, Sichuan | 99.76 | 29 | 20 | 87.79 | -1.9 | 28.5 | 100 |
| XCR | Ranwu Hot Spring, Xiangcheng, Sichuan | 99.84 | 28.68 | 21 | 84.65 | -5.83 | 27.3 | 94 |
| XG | Xianggelila, Yunnan | 99.91 | 27.8 | 12 | 70.55 | -2.22 | 26.1 | 91 |
| XCW | Wuming Mts., Xiangcheng, Sichuan | 100.02 | 29.12 | 18 | 93.33 | -7.72 | 30.2 | 103 |
| LJX | Xionggu, Lijiang, Yunnan | 100.03 | 26.86 | 10 | 60.14 | 7.6 | 23.4 | 97 |
| LJ | Alpine Botanic Garden, Lijiang, Yunnan | 100.19 | 27 | 16 | 67.4 | 2.58 | 25.4 | 96 |
| LJB | Baisha, Lijiang, Yunnan | 100.26 | 26.94 | 21 | 66.14 | 4.4 | 24.9 | 97 |
| DCK | Kalong, Daocheng, Sichuan | 100.26 | 28.68 | 24 | 88.32 | -4.35 | 28.2 | 111 |
| DCE | Eluo, Daocheng, Sichuan | 100.28 | 28.95 | 21 | 93.1 | -5.18 | 30.9 | 112 |
| YJH | Honglong, Yajiang, Sichuan | 100.69 | 30.15 | 22 | 104.73 | -5.62 | 31.1 | 131 |
| YJX | Yajiang, Sichuan | 100.95 | 30.01 | 15 | 102.52 | -0.95 | 28.6 | 146 |
| DFL | Longdeng, Daofu, Sichuan | 101.12 | 30.08 | 10 | 100.87 | 0.9 | 27.8 | 138 |
| DFY | Yinen, Daofu, Sichuan | 101.15 | 31.44 | 11 | 93.41 | -3.38 | 30.3 | 138 |
| RT | Rangtang, Sichuan | 101.24 | 31.79 | 9 | 91.72 | -4.38 | 30.3 | 134 |
| DFG | Geka, Daofu, Sichuan | 101.3 | 30.79 | 8 | 96.46 | -6.1 | 31.2 | 142 |
| KDD | Zheduo Mts., Kangding, Sichuan | 101.42 | 30.07 | 6 | 99.67 | -3.02 | 31.3 | 153 |
| MEKR | Songgang, Maerkang, Sichuan | 101.46 | 31.78 | 7 | 90.78 | -1.4 | 29.8 | 138 |
| DMX | Mingxi, Kangding, Sichuan | 101.5 | 29.71 | 16 | 99.1 | -1.45 | 29.5 | 158 |
| YYP | Pingchuanyakou, Yanyuan, Sichuan | 101.71 | 27.53 | 23 | 89.65 | 0.08 | 26.7 | 157 |
| KDC | Zhonggu, Kangding, Sichuan | 101.89 | 30.24 | 11 | 94.09 | -0.53 | 28.6 | 156 |
| KDZ | Zheduotang, Kangding, Sichuan | 101.9 | 29.99 | 10 | 94.51 | -1.52 | 28.7 | 161 |
| JCD | Dugang Mts., Jinchuan, Sichuan | 102.04 | 31.48 | 9 | 87.27 | 2.13 | 30 | 132 |
| JCK | Kasa, Jinchuan, Sichuan | 102.06 | 31.27 | 14 | 87.44 | 3.7 | 29.9 | 129 |
| MEKD | Dalangjiaogou, Maerkang, Sichuan | 102.12 | 31.91 | 20 | 86.42 | 0.03 | 30.8 | 129 |
| XJX | Xiamozi, Xiaojin, Sichuan | 102.39 | 30.9 | 10 | 88.57 | -1.27 | 27.6 | 146 |
| MEKS | Suomo, Maerkang, Sichuan | 102.51 | 31.86 | 10 | 83.92 | -3.3 | 29.9 | 133 |
| XJD | Dawei, Xiaojin, Sichuan | 102.52 | 30.88 | 13 | 87.89 | -8.05 | 29.8 | 152 |
| LX | Lixian, Sichuan | 102.82 | 31.65 | 10 | 81.45 | -3.98 | 32.7 | 129 |
| HS | Heishui, Sichuan | 102.93 | 32.08 | 11 | 80.08 | -1.22 | 32.6 | 124 |
| MX | Maoxian, Sichuan | 103.52 | 31.77 | 13 | 79.36 | -2.87 | 32.4 | 123 |
| WC | Wenchuan, Sichuan | 103.67 | 31.49 | 11 | 77.11 | -0.08 | 29.9 | 129 |
| PW | Pingwu, Sichuan | 104.53 | 32.46 | 10 | 94.55 | 4.8 | 29.3 | 105 |
| ***Quercus spinosa*** | | | | | | | | |
| TM | Tongmai, Bomi, Tibet | 95.1 | 30.1 | 5 | 91.42 | 4.93 | 27.6 | 171 |
| CYXC | Chayu, Tibet | 97.46 | 28.66 | 20 | 86.88 | 4.38 | 27.2 | 220 |
| BLLK | Bailonglaka, Chayu, Tibet | 98.12 | 28.58 | 15 | 73.39 | -3.77 | 27.4 | 101 |
| GZ | Alpine Botanical Garden, Xianggelila, Yunnan | 99.63 | 27.91 | 5 | 67.32 | -0.82 | 27.5 | 87 |
| XGJQ | Xionggu, Lijiang, Yunnan | 100.04 | 26.86 | 5 | 61.36 | 6.97 | 23.6 | 95 |
| GSL | Gesala, Yanbian, Sichuan | 101.26 | 27.17 | 9 | 82.71 | 3.33 | 25.4 | 138 |
| SS | Shun Mts., Jiulong, Sichuan | 101.51 | 29.01 | 9 | 99.08 | 2.87 | 27.3 | 191 |
| SWJ | Sawajiao, Jinchuan, Sichuan | 101.92 | 31.57 | 11 | 88.81 | 1 | 29.3 | 134 |
| GDS | Shun Mts., Kangding, Sichuan | 101.98 | 30.06 | 11 | 92.9 | 1.37 | 27.4 | 157 |
| KRC | Shun Mts., Jiulong, Sichuan | 102.05 | 30.67 | 10 | 89.03 | 3.7 | 28.4 | 137 |
| ELM | Erlangmiao, Luding, Sichuan | 102.23 | 29.9 | 8 | 91.36 | 5.38 | 27.4 | 162 |
| SMX | Suomo, Maerkang, Sichuan | 102.48 | 31.87 | 9 | 83.68 | -2.4 | 30.3 | 132 |
| BQX | Banqiao, Yuexi, Sichuan | 102.53 | 28.74 | 14 | 92.53 | 4.62 | 27.7 | 177 |
| LTS | Longtang Mts., Hanyuan, Sichuan | 102.62 | 29.3 | 13 | 89.59 | 4.12 | 27.8 | 168 |
| FTZ | Fengtongzhai., Baoxing, Sichuan | 102.87 | 30.57 | 14 | 79.67 | 2.15 | 29.7 | 151 |
| SLX | Sanlong, Maoxian, Sichuan | 103.54 | 31.79 | 12 | 79.79 | -1.62 | 33.5 | 121 |
| LBZ | Luobozhai, Wenchuan, Sichuan | 103.68 | 31.5 | 10 | 79.98 | 2.17 | 29.1 | 124 |
| JZG | Jiuzhaigou, Sichuan | 104.29 | 33.25 | 16 | 81.55 | -1.88 | 33.3 | 93 |
| ZGF | Zuigaofeng, Weining, Guizhou | 104.48 | 26.97 | 19 | 83.34 | 1.37 | 24.2 | 188 |
| CBLC | Changba Forest Farm, Kangxian, Sichuan | 105.49 | 33.38 | 13 | 81.22 | 0.07 | 29.8 | 90 |
| JFS | Jifeng Mts., Gansu | 105.68 | 33.69 | 14 | 79.53 | 1.13 | 29.1 | 89 |
| SMJQ | Shimen, Maiji, Gansu | 106.1 | 34.41 | 10 | 79.22 | -3.33 | 32.3 | 78 |
| WLD | Wulongdong, Lueyang, Shaanxi | 106.29 | 33.6 | 20 | 77.44 | -1.87 | 30 | 91 |
| JJY | Jiujiangyu, Dongcha, Gansu | 106.54 | 34.35 | 10 | 79.06 | -0.58 | 30.5 | 72 |
| ZJH | Zhangjiahe, Mianxian, Shaanxi | 106.57 | 33.36 | 21 | 77.12 | -0.98 | 30.1 | 99 |
| XLS | Xianglu Mts., Nanjiang, Sichuan | 107 | 32.72 | 15 | 73.62 | -2.63 | 31 | 118 |
| XHS | Xiaohua Mts., Taibai, Shaanxi | 107.3 | 34.01 | 21 | 75.79 | -4.12 | 32.3 | 83 |
| LC | Longchi Forest Farm, Xixiang, Shaanxi | 107.45 | 32.66 | 21 | 70.72 | -0.78 | 29.4 | 117 |
| HHG | Honghegu, Meixian, Shaanxi | 107.75 | 34.11 | 15 | 75.12 | -0.3 | 32.3 | 71 |
| TJS | Tiejiashu, Zhouzhi, Shaanxi | 107.83 | 33.88 | 9 | 73.88 | -3.07 | 32.1 | 85 |
| ZQ | Zhuque Forest Park, Huxian, Shaanxi | 108.54 | 33.81 | 8 | 71.92 | -1.8 | 31 | 83 |
| MWS | Muwang Mts., Zhenan, Shaanxi | 108.63 | 33.41 | 20 | 71.16 | -3.08 | 30.2 | 104 |
| NBL | Niubeiliang, Zhashui, Shaanxi | 108.87 | 33.8 | 22 | 71.7 | -2.62 | 31.9 | 87 |
| NGS | Nangong Mts., Langao, Shaanxi | 109.08 | 32.25 | 20 | 66.15 | 0.02 | 28.6 | 136 |
| BDX | Badaoxiang, Pingli, Shaanxi | 109.29 | 32.15 | 11 | 65.88 | -0.63 | 29.8 | 138 |
| TSX | Tianshuxia, Pingli, Shaanxi | 109.3 | 32.02 | 21 | 65.07 | -3.07 | 29.8 | 148 |
| PTS | Piantou Mts., Zhuxi, Hubei | 109.66 | 32.26 | 15 | 66.93 | 2.72 | 29.3 | 129 |
| BSY | Baishanyuan, Shennongjia, Hubei | 110.38 | 31.64 | 22 | 64.1 | -1.6 | 29.4 | 163 |
| DJP | Duanjiangping, Shennongjia, Hubei | 110.4 | 31.51 | 22 | 62.87 | -2.73 | 29.5 | 168 |
| TMS | Tianmen Mts., Zhangjiajie, Hubei | 110.48 | 29.05 | 12 | 57.08 | 4.9 | 29.5 | 242 |
| TDY | Tudiya, Shennongjia, Hubei | 110.51 | 31.8 | 20 | 64.46 | -1.45 | 29.1 | 162 |
| DLL | Dalaoling, Zigui, Hubei | 110.94 | 31.07 | 20 | 60.24 | 0.3 | 28.9 | 179 |
| JLT | Jiulingtou, Zigui, Hubei | 110.98 | 30.82 | 20 | 66 | 6.45 | 30.2 | 146 |
| WDS | Wudang Mts., Shiyan, Hubei | 111.07 | 32.47 | 20 | 65.77 | 4.17 | 33.4 | 113 |
| WKS | Wukuang Mts., Wuning, Jiangxi | 114.96 | 28.12 | 15 | 55.64 | 8.78 | 32.1 | 255 |
| SQS | Sanqing Mts., Yushan, Jiangxi | 118.06 | 28.91 | 9 | 53.84 | 3.48 | 26.7 | 365 |

**Table S2.** Description and reference of 25 microsatellites used for the initial amplification of *Q. aquifolioides* and *Q. spinosa*.

| Locus | Ta(°C) | Motif | Primer Sequence (5’ to 3’) | Successfully amplification | Allele range (bp) | Ref |
| --- | --- | --- | --- | --- | --- | --- |
| MSQ13 | 56 | TC | TGGCTGCACCTATGGCTCTTAG | No | 188-226 | Dow et al., 1995 |
|  |  |  | ACACTCAGACCCACCATTTTTCC |  |  |  |
| QpZAG16 | 56 | TC | CTTCACTGGCTTTTCCTCCT | **Yes** | 131-189 | Steinkellner et al., 1997 |
|  |  |  | TGAAGCCCTTGTCAACATGC |  |  |  |
| QrZAG7 | 56 | AG | CAACTTGGTGTTCGGATCAA | No | 108-154 | Kampfer et al., 1998 |
|  |  |  | GTGCATTTCTTTTATAGCATTCAC |  |  |  |
| QrZAG11 | 56 | AG | CCTTGAACTCGAAGGTGTCCTT | No | 241-283 | Kampfer et al., 1998 |
|  |  |  | GTAGGTCAAAACCATTGGTTGACT |  |  |  |
| QrZAG30 | 56 | GA | TGCTCCGTCATAATCTTGCTCTGA | **Yes** | 160-208 | Kampfer et al., 1998 |
|  |  |  | GCAATCCTATCATGCACATGCACAT |  |  |  |
| QrZAG87 | 56 | TC | TCCCACCACTTTGGTCTCTCA | No | 99-123 | Kampfer et al., 1998 |
|  |  |  | GTTGTC AGCAGTGGGATGGGTA |  |  |  |
| QrZAG96 | 56 | AG | CCCAGTCACATCCACTACTGTCC | No | 160-238 | Kampfer et al., 1998 |
|  |  |  | GGTTGGGAAAAGGAGATCAGA |  |  |  |
| PIE271 | 56 | AG | CACACTCACCAACCCTACCC | **Yes** | 197-247 | Durand et al., 2010 |
|  |  |  | GTGCGGTTGTAGACGGAGAT |  |  |  |
| QpZAG9 | 56 | TG | GCAATTACAGGCTAGGCTGG | **Yes** | 220-274 | Steinkellner et al., 1997 |
|  |  |  | GTCTGGACCTAGCCCTCATG |  |  |  |
| QpZAG110 | 56 | AG | GGAGGCTTCCTTCAACCTACT | **Yes** | 193-267 | Steinkellner et al., 1997 |
|  |  |  | GATCTCTTGTGTGCTGTATTT |  |  |  |
| QrZAG 112 | 56 | GA | TTCTTGCTTTGGTGCGCG | **Yes** | 98-116 | Kampfer et al., 1998 |
|  |  |  | GTGGTCAGAG ACTCGGTAAGTATTC |  |  |  |
| QmC02241 | 56 | GA/TC | TCAGTGACCACACGTCACCTCTC | **Yes** | 169-211 | Ueno et al., 2008 |
|  |  |  | GTTTCTTGGCCATGTTTTGATGG |  |  |  |
| CN725667 | 56 | CCA/TTC | GCTAAGCTCCAAGCCATTTGTGA | No | 194-272 | Ueno et al., 2008 |
|  |  |  | GTTTCCGATGACGTGGATGTAATCTCC |  |  |  |
| CR627959 | 56 | GA/TGC | GCTCCCTGGTAGTCGGCTAAAGA | No | 232-296 | Ueno et al., 2008 |
|  |  |  | GTTTCAATTGGGACAACATGGAAGCAT |  |  |  |
| GOT011 | 56 | GA | CCCCACCGTCTACTCTCAAA | **Yes** | 197-225 | Durand et al., 2010 |
|  |  |  | GCGTTCACCACGTCCATAAT |  |  |  |
| GOT012 | 56 | GT | TGATGATCCCAAACCACAAA | **Yes** | 204-254 | Durand et al., 2010 |
|  |  |  | AAGGCTGCAGGACTTTTCAA |  |  |  |
| GOT021 | 56 | AT | AGAAAGTTCCAGGGAAAGCA | **Yes** | 110-150 | Durand et al., 2010 |
|  |  |  | CTTCGTCCCCAGTTGAATGT |  |  |  |
| GOT040 | 56 | TC | AAGGCACTCGTCGCTTTCTA | **Yes** | 242-298 | Durand et al., 2010 |
|  |  |  | ACCGATTTGAAGCTCGAGAA |  |  |  |
| PIE163 | 56 | TC | GAGAGGCATGTGGAACCAAG | **Yes** | 230-264 | Durand et al., 2010 |
|  |  |  | CAAGCATAGGTGGTGGAACC |  |  |  |
| FIR026 | 56 | GT/GA | CTTCATGCACCAATTCCTCA | **Yes** | 201-215 | Durand et al., 2010 |
|  |  |  | GGCCATGTATGTGTGCAAAA |  |  |  |
| WAG066 | 56 | TC | AACCTGTTTGGCTTCGTGTG | No | 128-224 | Durand et al., 2010 |
|  |  |  | AACAAAAGATTGGGAGGTGC |  |  |  |
| WAG068 | 56 | TC/TG | TCTGCAACAAAACCAAAACAC | No | 154-210 | Durand et al., 2010 |
|  |  |  | CGGAGGAGAGAGTCAGCAAC |  |  |  |
| POR017 | 56 | GA | CCCATATCCCTCTACGAAAGAA | No | 126-170 | Durand et al., 2010 |
|  |  |  | CTGGAGATGACATAGTGTCTCAAA |  |  |  |
| POR025 | 56 | AG | CACACAAACCCATATGATCTGAA | **Yes** | 105-145 | Durand et al., 2010 |
|  |  |  | TCTCTTTCGATCCCTTCTGC |  |  |  |
| FIR015 | 56 | GT | ACCCTAAAACCCCAATCACC | **Yes** | 112-148 | Durand et al., 2010 |
|  |  |  | CGGATCTTCGGCTATTCTTG |  |  |  |

**Cited references**

Dow, B.D., Ashley, M.V. & Howe H.F. (1995) Characterization of highly variable (GA/CT)n microsatellites in the bur oak, Quercus macrocarpa. Theoretical & Applied Genetics, 91, 137–141.

Kampfer S, Lexer C, Glössl J, Steinkellner H. (1998). Characterization of (GA)n microsatellite loci from *Quercus robur*. Hereditas, 129(2): 183-186.

Durand J, Bodénès C, Chancerel E, de Daruvar A, Kremer A, Plomion C. (2010). A fast and cost-effective approach to develop and map EST-SSR markers: oak as a case study. BMC Genomics, 11(1): 570.

Steinkellner H, Fluch S, Turetschek E, Lexer C, Streiff R, Kremer A, Burg K, Glossl J. (1997). Identification and characterization of (GA/CT)n-microsatellite loci from Quercus petraea. Plant Molecular Biology, 33(6): 1093-1096.

Ueno S, Taguchi Y, Tsumura Y.. (2008). Microsatellite markers derived from *Quercus mongolica* var. *crispula* (Fagaceae) inner bark expressed sequence tags. Genes & Genetic Systems, 83(2): 179-187.

**Table S3.** Genetic diversity estimates for SSR datasets of *Q. aquifolioides* and *Q. spinosa*. *H*_O_, observed heterozygosity; *H*_E_, expected heterozygosity; *N*_E_, effective population size; *I*, Shannon index.

| **code** | ***N*_E_** | ***I*** | ***H*_O_** | ***H*_E_** | ***uH*_E_** |
| --- | --- | --- | --- | --- | --- |
| ***Quercus aquifolioides*** | | | | |  |
| *Q.aqu*-West lineage | | | | |  |
| LZD | 2.91 | 1.07 | 0.53 | 0.53 | 0.54 |
| GBX | 3.09 | 1.11 | 0.5 | 0.54 | 0.55 |
| MLJ | 2.65 | 0.93 | 0.46 | 0.47 | 0.48 |
| GB | 2.81 | 1.05 | 0.54 | 0.53 | 0.49 |
| MLL | 2.87 | 1.11 | 0.54 | 0.53 | 0.54 |
| SJLS | 2.08 | 0.81 | 0.49 | 0.44 | 0.48 |
| DZC | 2.55 | 0.9 | 0.53 | 0.47 | 0.56 |
| KDG | 2.61 | 0.97 | 0.6 | 0.51 | 0.52 |
| LZA | 2.88 | 1.08 | 0.53 | 0.54 | 0.55 |
| BYZ | 2.68 | 1.03 | 0.59 | 0.52 | 0.54 |
| LZ | 2.96 | 1.02 | 0.58 | 0.54 | 0.56 |
| BM | 3.04 | 1.11 | 0.51 | 0.57 | 0.59 |
| LL | 2.66 | 1.01 | 0.53 | 0.52 | 0.54 |
| MLP | 3.07 | 1.13 | 0.55 | 0.57 | 0.58 |
| BMS | 2.83 | 0.97 | 0.5 | 0.49 | 0.50 |
| BMR | 2.83 | 1.02 | 0.52 | 0.52 | 0.53 |
| BMZ | 2.74 | 1.02 | 0.49 | 0.53 | 0.56 |
| mean | 2.78 | 1.02 | 0.53 | 0.52 | 0.54 |
| *Q.aqu*-East lineage | | | | |  |
| CY | 3.01 | 1.14 | 0.57 | 0.56 | 0.59 |
| CYX | 2.81 | 1.04 | 0.54 | 0.53 | 0.55 |
| CBG | 2.91 | 1.13 | 0.56 | 0.54 | 0.56 |
| MKD | 3.42 | 1.32 | 0.6 | 0.65 | 0.67 |
| MKR | 3.32 | 1.25 | 0.56 | 0.6 | 0.61 |
| MKZ | 3.44 | 1.29 | 0.6 | 0.63 | 0.65 |
| DQ | 3.53 | 1.29 | 0.56 | 0.62 | 0.64 |
| BZL | 3.02 | 1.22 | 0.59 | 0.6 | 0.62 |
| DRR | 3.52 | 1.29 | 0.55 | 0.61 | 0.62 |
| DRX | 3.32 | 1.25 | 0.57 | 0.6 | 0.61 |
| ZD | 3.46 | 1.29 | 0.64 | 0.63 | 0.65 |
| XCD | 3.4 | 1.24 | 0.53 | 0.59 | 0.60 |
| XCR | 3.24 | 1.29 | 0.63 | 0.62 | 0.64 |
| XG | 3.42 | 1.2 | 0.53 | 0.58 | 0.61 |
| XCW | 3.54 | 1.31 | 0.6 | 0.64 | 0.66 |
| LJX | 2.78 | 1.01 | 0.53 | 0.51 | 0.54 |
| LJ | 3.36 | 1.21 | 0.56 | 0.58 | 0.60 |
| DCK | 3.6 | 1.33 | 0.58 | 0.63 | 0.62 |
| LJB | 3.35 | 1.28 | 0.52 | 0.6 | 0.66 |
| DCE | 3.35 | 1.28 | 0.54 | 0.62 | 0.64 |
| YJH | 3.24 | 1.29 | 0.59 | 0.63 | 0.66 |
| YJX | 3.17 | 1.2 | 0.55 | 0.59 | 0.61 |
| DFL | 2.86 | 1.17 | 0.6 | 0.6 | 0.64 |
| DFY | 3.52 | 1.24 | 0.61 | 0.62 | 0.66 |
| RT | 3.1 | 1.25 | 0.64 | 0.64 | 0.68 |
| DFG | 3.27 | 1.24 | 0.68 | 0.63 | 0.68 |
| KDD | 2.95 | 1.14 | 0.66 | 0.59 | 0.65 |
| MEKR | 3.05 | 1.17 | 0.62 | 0.61 | 0.67 |
| DMX | 3.31 | 1.26 | 0.66 | 0.62 | 0.63 |
| YYP | 3.28 | 1.25 | 0.57 | 0.6 | 0.61 |
| KDC | 3.32 | 1.24 | 0.62 | 0.61 | 0.65 |
| KDZ | 3.54 | 1.35 | 0.67 | 0.65 | 0.69 |
| JCD | 3.3 | 1.27 | 0.7 | 0.65 | 0.69 |
| JCK | 3.52 | 1.28 | 0.64 | 0.63 | 0.65 |
| MEKD | 3.66 | 1.35 | 0.64 | 0.65 | 0.68 |
| XJX | 3.26 | 1.23 | 0.57 | 0.62 | 0.66 |
| MEKS | 3.66 | 1.32 | 0.66 | 0.64 | 0.68 |
| XJD | 3.53 | 1.3 | 0.64 | 0.64 | 0.67 |
| LX | 3.35 | 1.31 | 0.62 | 0.65 | 0.69 |
| HS | 3.23 | 1.25 | 0.64 | 0.63 | 0.67 |
| MX | 3.5 | 1.33 | 0.69 | 0.66 | 0.69 |
| WC | 3.58 | 1.31 | 0.63 | 0.63 | 0.66 |
| PW | 3.57 | 1.29 | 0.65 | 0.62 | 0.66 |
| mean | 3.31 | 1.25 | 0.6 | 0.61 | 0.64 |
| *P* | <0.01 | <0.01 | <0.01 | <0.01 | <0.01 |
| mean total | 3.16 | 1.18 | 0.59 | 0.58 | 0.61 |
| ***Quercus spinosa*** | | | | |  |
| *Q.spi*-West lineage | | | | |  |
| TM | 2.34 | 0.85 | 0.41 | 0.51 | 0.56 |
| CYXC | 2.39 | 0.81 | 0.26 | 0.41 | 0.42 |
| BLLK | 2.75 | 0.98 | 0.32 | 0.48 | 0.50 |
| GZ | 2.56 | 0.96 | 0.39 | 0.53 | 0.58 |
| XGJQ | 1.97 | 0.7 | 0.23 | 0.4 | 0.45 |
| GSL | 2.53 | 0.92 | 0.28 | 0.48 | 0.51 |
| SS | 2.36 | 0.82 | 0.35 | 0.43 | 0.45 |
| SWJ | 3.07 | 1.05 | 0.5 | 0.51 | 0.53 |
| GDS | 2.82 | 1.05 | 0.47 | 0.52 | 0.54 |
| KRC | 2.86 | 1.09 | 0.51 | 0.54 | 0.57 |
| ELM | 2.12 | 0.78 | 0.46 | 0.43 | 0.46 |
| SMX | 2.56 | 0.96 | 0.5 | 0.52 | 0.55 |
| BQX | 2.16 | 0.83 | 0.35 | 0.44 | 0.45 |
| LTS | 2.33 | 0.79 | 0.37 | 0.4 | 0.42 |
| FTZ | 2.69 | 0.97 | 0.5 | 0.5 | 0.52 |
| SLX | 2.65 | 0.94 | 0.52 | 0.48 | 0.50 |
| LBZ | 2.82 | 1.07 | 0.51 | 0.55 | 0.57 |
| JZG | 2.16 | 0.8 | 0.35 | 0.41 | 0.43 |
| ZGF | 2.55 | 1.01 | 0.52 | 0.51 | 0.52 |
| CBLC | 2.43 | 0.84 | 0.43 | 0.44 | 0.45 |
| JFS | 2.15 | 0.81 | 0.31 | 0.43 | 0.44 |
| SMJQ | 2.37 | 0.91 | 0.41 | 0.47 | 0.49 |
| mean | 2.48 | 0.91 | 0.41 | 0.47 | 0.50 |
| *Q.spi-East* lineage | | | | |  |
| WLD | 3.24 | 1.2 | 0.54 | 0.59 | 0.61 |
| JJY | 2.3 | 0.83 | 0.43 | 0.44 | 0.46 |
| ZJH | 3.16 | 1.18 | 0.51 | 0.6 | 0.61 |
| XLS | 2.3 | 0.92 | 0.42 | 0.47 | 0.49 |
| XHS | 3.15 | 1.19 | 0.52 | 0.59 | 0.60 |
| LC | 2.33 | 0.96 | 0.52 | 0.52 | 0.53 |
| HHG | 2.53 | 1.02 | 0.37 | 0.55 | 0.57 |
| TJS | 2.04 | 0.78 | 0.34 | 0.44 | 0.47 |
| ZQ | 2.61 | 0.96 | 0.41 | 0.51 | 0.54 |
| MWS | 3.28 | 1.2 | 0.48 | 0.6 | 0.62 |
| NBL | 2.84 | 1.08 | 0.5 | 0.55 | 0.57 |
| NGS | 2.77 | 1.06 | 0.48 | 0.54 | 0.55 |
| BDX | 2.56 | 1.04 | 0.61 | 0.56 | 0.59 |
| TSX | 2.89 | 1.11 | 0.46 | 0.57 | 0.59 |
| PTS | 2.03 | 0.76 | 0.3 | 0.4 | 0.41 |
| BSY | 2.57 | 1.06 | 0.42 | 0.54 | 0.55 |
| DJP | 2.59 | 0.97 | 0.36 | 0.49 | 0.50 |
| TMS | 2.61 | 1.01 | 0.56 | 0.53 | 0.55 |
| TDY | 2.32 | 0.84 | 0.29 | 0.42 | 0.43 |
| DLL | 2.98 | 1.08 | 0.31 | 0.55 | 0.56 |
| JLT | 2.6 | 0.93 | 0.32 | 0.47 | 0.48 |
| WDS | 2.22 | 0.89 | 0.34 | 0.48 | 0.49 |
| WKS | 2.41 | 0.94 | 0.28 | 0.51 | 0.53 |
| SQS | 2.16 | 0.76 | 0.34 | 0.42 | 0.45 |
| mean | 2.6 | 0.99 | 0.42 | 0.51 | 0.53 |
| *P* | <0.01 | <0.01 | <0.01 | <0.01 | <0.01 |
| Mean total | 2.55 | 0.95 | 0.41 | 0.49 | 0.52 |

**Table S4.** Mantel test and partial Mantel test (conditioned with geographical distance) between pairwise genetic distance (*F*_ST_ / (1 - *F*_ST_)) and four environmental distances in different lineages and all populations of *Q. aquifolioides* and *Q. spinosa.*

|  | ***Q. aquifolioides*** | |  | ***Q. aqu*-West lineage** | |  | ***Q. aqu*-East lineage** | |  | ***Q. spinosa*** | |  | ***Q. spi*-West lineage** | |  | ***Q. spi*-East lineage** | |
| --- | --- | --- | --- | --- | --- | --- | --- | --- | --- | --- | --- | --- | --- | --- | --- | --- | --- |
|  | **Mantel’s *r*** | ***P*** |  | **Mantel’s *r*** | ***P*** |  | **Mantel’s *r*** | ***P*** |  | **Mantel’s *r*** | ***P*** |  | **Mantel’s *r*** | ***P*** |  | **Mantel’s *r*** | ***P*** |
| *Mantel test* | | | | |  |  |  |  |  |  |  |  |  |  |  |  |  |
| bio07 | 0.36 | **<0.001** |  | -0.22 | 0.951 |  | 0.49 | **<0.001** |  | 0.21 | **0.005** |  | 0.23 | 0.063 |  | 0.15 | 0.155 |
| bio09 | -0.01 | 0.601 |  | 0.04 | 0.373 |  | -0.02 | 0.781 |  | 0.02 | 0.274 |  | 0.08 | 0.109 |  | 0.04 | 0.268 |
| bio15 | 0.27 | **0.003** |  | -0.05 | 0.609 |  | 0.33 | **0.004** |  | 0.44 | **<0.001** |  | 0.21 | **0.04** |  | 0.42 | **0.001** |
| prec06 | 0.03 | 0.264 |  | -0.08 | 0.661 |  | 0.13 | 0.083 |  | 0.19 | **0.003** |  | 0.2 | **0.019** |  | 0.42 | **0.002** |
| *partial Mantel test* | | | | | |  |  |  |  |  |  |  |  |  |  |  |  |
| bio07 | 0.32 | **<0.001** |  | -0.23 | 0.961 |  | 0.41 | **<0.001** |  | -0.01 | 0.533 |  | 0.08 | 0.247 |  | -0.08 | 0.72 |
| bio09 | 0.01 | 0.554 |  | 0.03 | 0.404 |  | -0.01 | 0.606 |  | 0.02 | 0.225 |  | 0.13 | **0.002** |  | 0.06 | 0.188 |
| bio15 | 0.26 | **0.004** |  | -0.19 | 0.867 |  | 0.29 | **0.01** |  | 0.18 | **0.003** |  | 0.18 | 0.092 |  | -0.04 | 0.625 |
| prec06 | 0.03 | 0.31 |  | -0.19 | 0.854 |  | -0.01 | 0.461 |  | -0.02 | 0.605 |  | 0.08 | 0.149 |  | -0.01 | 0.506 |

**Table S5.** Correlations (Spearman’s ρ) of genetic distance (*F*_ST_ / (1 - *F*_ST_)) with geographical & environmental distances in different lineages and all populations of *Q. aquifolioides* and *Q. spinosa* estimated by MRM (Multiple Regression on distance Matrices).

|  | ***Q. aquifolioides*** | |  | ***Q. aqu*-West lineage** | |  | ***Q. aqu*-East lineage** | |  | ***Q. spinosa*** | |  | ***Q. spi*-West lineage** | |  | ***Q. spi*-East lineage** | |
| --- | --- | --- | --- | --- | --- | --- | --- | --- | --- | --- | --- | --- | --- | --- | --- | --- | --- |
|  | **ρ** | ***P*** |  | **ρ** | ***P*** |  | **ρ** | ***P*** |  | **ρ** | ***P*** |  | **ρ** | ***P*** |  | **ρ** | ***P*** |
| geographical distance | 0.57 | **<0.001** |  | 0.41 | <0.001 |  | 0.39 | 0.053 |  | 0.49 | **<0.001** |  | 0.24 | 0.035 |  | 0.54 | **0.007** |
| bio07 | 0.13 | **0.004** |  | -0.21 | 0.082 |  | 0.21 | **0.001** |  | -0.01 | 0.862 |  | 0.07 | 0.584 |  | -0.08 | 0.496 |
| bio09 | 0.09 | 0.623 |  | 0.06 | 0.602 |  | 0.12 | 0.602 |  | 0.03 | 0.528 |  | 0.17 | 0.297 |  | 0.2 | 0.098 |
| bio15 | 0.02 | **0.006** |  | -0.11 | 0.595 |  | 0.04 | **0.025** |  | 0.16 | **0.002** |  | 0.12 | **0.02** |  | -0.23 | 0.223 |
| prec06 | -0.06 | 0.16 |  | -0.24 | 0.202 |  | -0.14 | 0.079 |  | -0.04 | 0.353 |  | 0.03 | **0.09** |  | 0.12 | 0.46 |
| ρ2 and *P* of best model | 0.39 | **<0.001** |  | 0.14 | 0.213 |  | 0.24 | **<0.001** |  | 0.35 | **<0.001** |  | 0.16 | **<0.001** |  | 0.29 | **<0.001** |

**Table S6**. Variable importance (%) of each environmental variables of Generalized Dissimilarity Model (GDM) correlated with geographic and environmental variables in different lineages and all populations of *Q. aquifolioides* and *Q. spinosa*.

|  | ***Q. aquifolioides*** | ***Q. aqu*-West** | ***Q. aqu*-East** |  | ***Q. spinosa*** | ***Q. spi*-West** | ***Q. spi*-East** |
| --- | --- | --- | --- | --- | --- | --- | --- |
| Geographic | 59.29 | 62.89 | 2.78 |  | 53.43 | 7.19 | 8.88 |
| bio07 | 5.99 | 30.61 | 12.68 |  | 0 | 0.92 | 0 |
| bio09 | 14.7 | 0 | 0 |  | 0.35 | 0 | 1.93 |
| bio15 | 0.08 | 0.27 | 36.22 |  | 0 | 30.8 | 0.04 |
| prec06 | 16.81 | 0.07 | 24.43 |  | 13.56 | 27.82 | 0 |
